# Influence of Fragmentation Techniques on the Biotic Aging of Test Microplastics

**DOI:** 10.1021/acs.est.6c03742

**Published:** 2026-08-28

**Authors:** Barbara Klun, Serena Ducoli, Mark Starin, Janja Novak, Filippo Vaccari, Edoardo Puglisi, Ula Rozman, Nataša Čelan Korošin, Gabriela Kalčíková, Stefania Federici

**Affiliations:** † Faculty of Chemistry and Chemical Technology, 112794 University of Ljubljana, Večna pot 113, Ljubljana 1000, Slovenia; ‡ Department of Mechanical and Industrial Engineering, 9297 University of Brescia & INSTM RU of Brescia, Via Branze 38, Brescia 25123, Italy; § 9371 Università Cattolica del Sacro Cuore, Department for Sustainable Process, Faculty of Agriculture, Food and Environmental Science (DiSTAS), Via Emilia Parmense 84, Piacenza 29122, Italy

**Keywords:** aging, biofilm, biofouling, degradation, fragmentation, plastic pollution

## Abstract

This study investigates how mechanical fragmentation techniques—ultracentrifugal and mixer milling—affect the biotic aging of polystyrene (PS) and polypropylene (PP) microplastics in freshwater, focusing on biofilm formation, microbial colonization, and changes in their physicochemical properties. Both polymer type and fragmentation methods significantly affected biofilm development. Mixer-milled PP supported greater biofilm accumulation than ultracentrifugally milled PP, while the latter exhibited higher chlorophyll *a* and extracellular polymeric substances levels, indicating increased phototrophic activity. Metagenomic analysis showed a conserved community dominated by *Brevibacillus*, with fragmentation-dependent shifts. *Brevibacillus* and *Clostridium* enriched in ultracentrifugally milled samples, while *Priestia* was associated with mixer-milled particles. *Bradyrhizobium* remained stable despite variations in chlorophyll levels, suggesting a fragmentation-dependent metabolic shift. Plastic-degrading gene profiles mirrored biofilm trends, with oxidative enzymes (laccases and peroxidases) predominating. Spectroscopic and microscopic analysis confirmed functionalization and morphological differences, while density and crystallinity measurements revealed structural changes associated with colonization. These results emphasize the importance of surface properties in the preparation of test materials for microplastic analysis, which depends on the type of polymer and fragmentation method and influences microbial attachment and aging processes. Selection of fragmentation techniques therefore affect microplastic behavior, interactions, and environmental fate.

## Graphical abstract

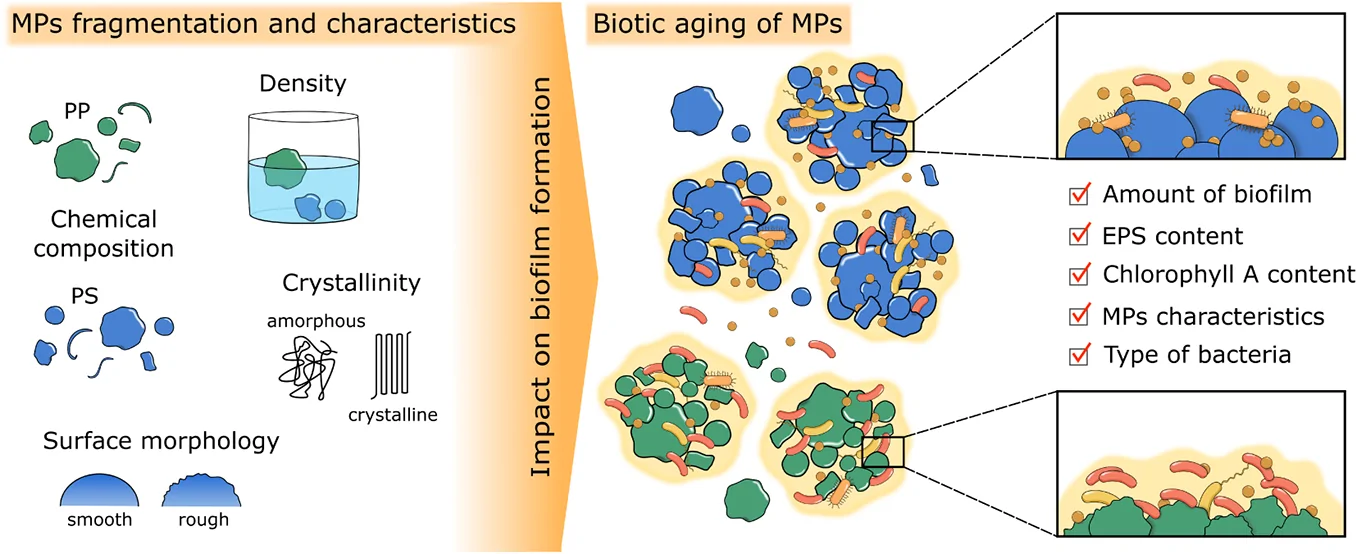

## 1 Introduction

Plastic pollution has emerged as a significant environmental issue over the past decade, with recent attention focused on the smaller fraction known as microplastics (MPs).^1^ MPs are already now found in diverse environments, including aquatic, terrestrial, and atmospheric systems, as well as in biota.^2,3^ The widespread presence of MPs in the environment, coupled with their persistence, underscores the urgency of understanding their long-term ecological and health impacts.

Regardless of their environmental compartments, MPs undergo aging processes that can significantly alter their chemical and physical properties. These aging processes are driven by both the biotic and abiotic factors. Biotic aging includes the formation of biofilms on particle surfaces, while abiotic aging involves physical mechanisms like UV light exposure, abrasion, and thermochemical oxidation.^4,5^ As MPs age, they can change their size, density, surface roughness, and shape.^6,7^ These modifications can have far-reaching consequences, such as altering their transport dynamics,^8,9^ changing capacity to adsorb pollutants,^7,10^ increasing the leaching of additives or chemicals,^11^ and altering their ecotoxicity.^10,12,13^ Therefore, understanding the effects of various aging processes on MPs is crucial for assessing their fate and ecological consequences.

Among these processes, biofilm formation plays a particularly important role because it directly mediates the interaction between MPs and aquatic ecosystems.^14^ The establishment of microbial communities on plastic surfaces can alter particle density and transport behavior, influence degradation pathways, modify the adsorption of contaminants, and affect interactions with organisms.^15,16^ Biofilm development is strongly influenced by surface-related properties of MPs, such as roughness, morphology, hydrophobicity, and surface chemistry.^17^ Consequently, the initial characteristics of MPs used in laboratory studies may substantially affect the observed biological responses.

Despite the growing body of research on MP aging and biofilm formation, limited attention has been paid to the influence of the manufacturing methods used to produce MPs as experimental test materials. These methods may substantially affect the resulting particle physicochemical properties and, consequently, their interactions with biological systems. Many laboratory studies rely on commercially available MP test materials, most commonly in the form of spherical particles,^18,19^ thereby limiting the ecological relevance, as the majority of MPs typically found in the environment are irregularly shaped.^20,21^ In fact, the aging of spherical MPs proceeds differently compared to environmentally relevant films and fragments.^22^ Therefore, it is valuable to prepare and use test materials that mimic MPs found in the environment. Top-down fragmentation appears to be the most relevant approach for producing test materials, as it more accurately reflects natural processes occurring in the environment compared to bottom-up approaches.^23^ However, recent insights into the comparison of fragmentation techniques revealed significant impacts on MP morphology, chemical composition, degree of crystallinity, and structural changes depending on the fragmentation method used.^24^ Nevertheless, the extent to which these fragmentation-induced differences influence biotic aging and biofilm formation remains largely unexplored. This issue is particularly relevant because mechanically fragmented MPs are widely used in ecotoxicological, analytical, and environmental fate studies, where they are often implicitly treated as interchangeable test materials. However, if fragmentation methods significantly alter microbial colonization and aging processes, then differences in particle preparation could contribute to variability among studies and potentially bias the experimental outcomes. Therefore, understanding how fragmentation techniques affect the physicochemical and biological behavior of MPs is essential for improving the comparability, reproducibility, and environmental relevance of MP research.

In this context, we aim to investigate how different fragmentation methods used to generate laboratory test MPs influence their subsequent biotic aging. We specifically compared two widely used mechanical fragmentation methods—ultracentrifugal milling and mixer milling—applied to polystyrene (PS) and polypropylene (PP) commercial plastics. These methods generate MPs through different mechanical forces and were previously shown to produce particles with distinct physicochemical characteristics.^24^ Here, we evaluated whether these fragmentation-induced differences affect: (i) biofilm formation, (ii) chlorophyll *a* content, (iii) content of extracellular polymeric substances (EPS) in the biofilm, (iv) microbial composition, and (v) changes in MP properties, including particle density, chemical composition, and surface morphology. The results of this study will enhance our understanding of how different fragmentation techniques influence the aging of MPs, providing valuable insights for the development of well-characterized test materials. By linking fragmentation techniques to biotic aging behavior, this study aims to demonstrate the importance of selecting and characterizing test materials for environmental MP research.

## 2 Materials and Methods

To provide a clear visual representation of the experimental workflow, Figure 1 illustrates the key steps involved in this study, highlighting the sequence of procedures and the methods used for analysis. The process began with the selection of the starting materials (daily used plastic items, with the polymeric content identified as PS and PP), followed by the fragmentation process using two different milling techniques: ultracentrifugal milling and mixer-milling. Following fragmentation, the MPs undergo biotic aging under controlled conditions, after which various characterizations are performed, including biofilm formation analysis, chlorophyll *a* and EPS content measurement, microbial composition assessment, and changes in the physicochemical properties of the MPs.

**Figure 1:**
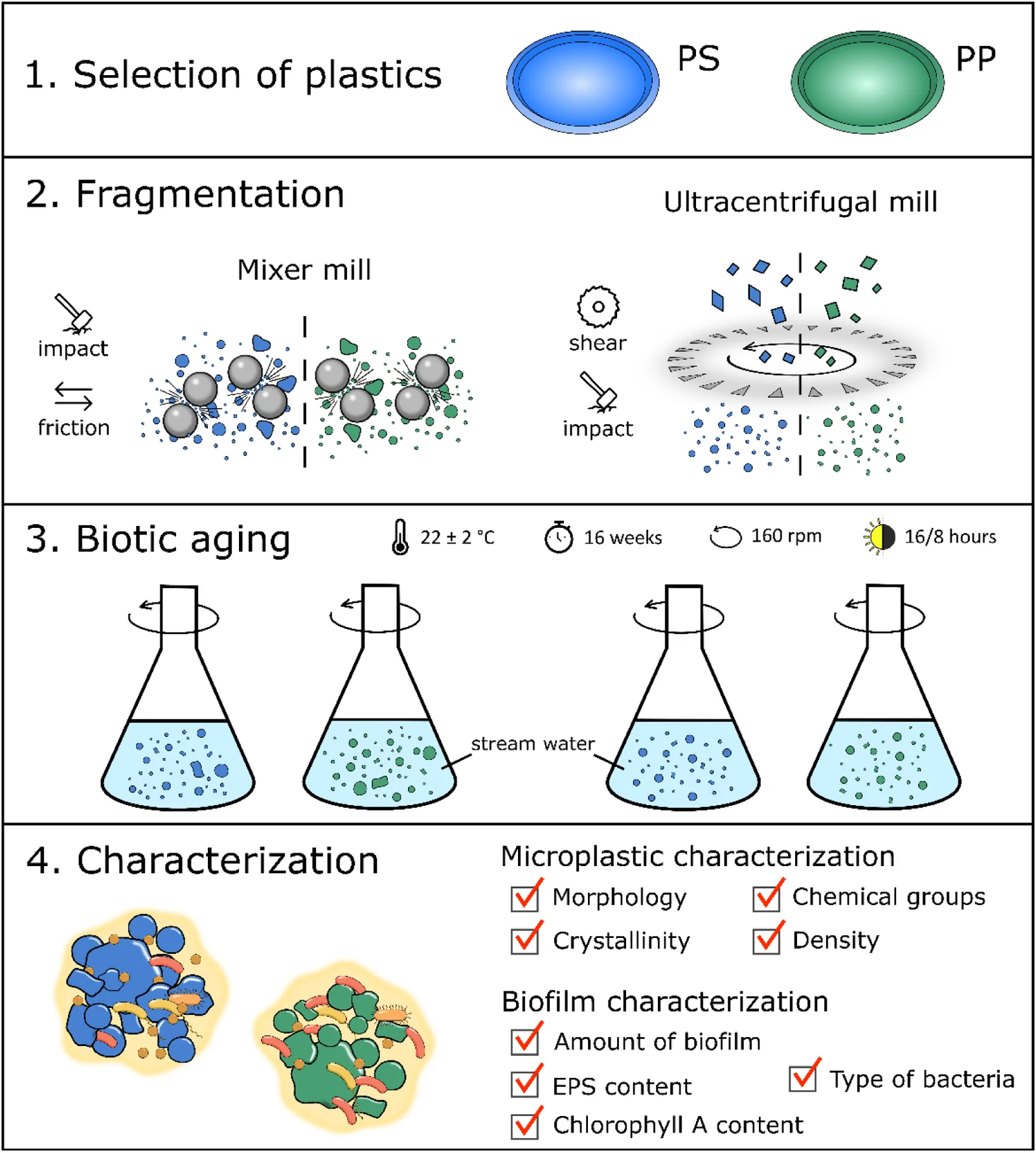
Experimental workflow. Overview of the fragmentation, biotic aging, biofilm formation, and characterization of microplastics.

### 2.1 Preparation of Test Microplastics

Two sets of commonly used plastic products made of the polymers polystyrene (PS) and polypropylene (PP), which are widely used in disposable and everyday items, were selected. Commercially available PS- and PP-based products were used as received without detailed information on dyes, additives, molecular weight, or purity to represent the plastics typically entering aquatic environments. These commercial plastics inherently contain complex and heterogeneous additive mixtures that are characteristic of environmentally relevant plastics and are not always fully identifiable. Since both fragmentation techniques were applied to the same starting material for each polymer type, any differences observed between treatments can be attributed primarily to fragmentation-induced physicochemical changes rather than differences in additive composition.

The study primarily aimed to compare the effects of different milling techniques on biofilm formation and microbial colonization. Two mechanical fragmentation techniques—ultracentrifugal milling and mixer milling—were used and compared to produce MPs, according to our previously developed protocols.^24^ These techniques rely on different mechanical forces to simulate the natural breakdown of plastics. Ultracentrifugal milling operates through impact and shear forces generated between a rapidly rotating rotor and a stationary ring sieve, while mixer milling operates through impact and friction forces due to the collision of plastic with the balls and the inner walls of the chamber.

These methods accelerate fragmentation to generate sufficient material for experiments and are commonly applied in MPs research.^25,26^ While not directly mimicking natural mechanical forces, they provide reproducible particle size distributions suitable for evaluating biofilm formation and microbial colonization. Blue PS disposable plates and green PP food containers were cut by hand with steel scissors, cleaned with Milli-Q water and a few drops of commercial detergent, and then sonicated in a water bath for 15 min to clean and remove oil and dirt from the pieces. After three washing cycles with Milli-Q water, the pieces were air-dried at room temperature. The plastic fragments were then embrittled in liquid nitrogen for 30 min. This preparation step is commonly used^25,27,28^ to produce MP test materials. For the ultracentrifugal milling protocol, the plastic pieces were placed in an ultracentrifugal mill (ZM 200, Retsch) cooled with liquid nitrogen. Under a constant stream of liquid nitrogen, the pieces were mixed at maximum speed (18,000 rpm) until they were completely fragmented. The mill included a sieve with a mesh size of 500 μm, which served as a fractionation stage. The powder was collected and stored for later analysis. For the mixer milling protocol, the plastic pieces were allowed to dry before being placed in 50 mL stainless steel grinding beakers with seven 12 mm diameter stainless steel balls that had previously been cooled in liquid nitrogen. A mixer mill (MM 400, Retsch) was used to grind the pieces for 10 min at a maximum frequency of 30 Hz. The resulting plastic powder was then sieved through a 500 μm filter and stored for further investigation.

Temperature increases during mechanical milling can potentially induce polymer degradation; however, in this study continuous cooling with liquid nitrogen was applied and FTIR analysis confirmed no detectable chemical changes in the fragmented polymers compared to pristine materials.^24^

### 2.2 Characterization of Test Microplastics

#### 2.2.1 Particle Size Distribution and Morphological Descriptors

The particle size distribution of pristine microplastics produced by the two fragmentation techniques was determined by using a laser diffraction analyzer (Microtrac S3500 Bluewave, USA) equipped with a dry dispersion unit. Measurements were performed in triplicate, and the resulting number-based size distributions were used for the data analysis.

Particle morphology was quantitatively characterized by an image analysis of optical micrographs. For each MP class (ultracentrifugally milled PS, mixer-milled PS, ultracentrifugally milled PP, and mixer-milled PP), high-magnification images of approximately 50 particles were acquired by using a stereomicroscope equipped with a digital camera (MZ16-A and MC190, Leica Microsystems, Germany). Particle descriptors were extracted using Leica Image Analysis software (Leica Application Suite (LAS) 4 software enhanced with interactive measurements and image analysis modules), including perimeter, convex perimeter, area, and convex area. Particle edge irregularity was assessed through convexity, defined as the ratio of the convex perimeter to the actual particle perimeter (convex perimeter/perimeter). A convexity value approaching 1 indicates particles with smooth, regular outlines, whereas lower values indicate increasingly irregular and jagged particle edges. In addition, solidity was calculated as the ratio between the particle area and the area of its convex hull (area/convex area), providing a descriptor of the overall compactness of the particle shape and the presence of large concavities.^29,30^

#### 2.2.2 Apparent Wettability of Microplastics

The apparent wettability of test MPs before the aging process was evaluated by static sessile drop contact angle measurements performed on compacted MP pellets. Approximately 150 mg of MPs was compressed into flat pellets using a hydraulic press (200 bar, 4 min). The pellets were stored under ambient laboratory conditions for 24 h prior to analysis to allow for equilibration.

Static contact angle measurements were performed at room temperature using a contact angle goniometer (CAM 200 tensiometer (KSV Instruments, Finland) equipped with a Navitar camera). Milli-Q water droplets (about 4 μL) were gently deposited onto the pellet surface, and droplet images were acquired using a high-resolution digital camera. Contact angles were determined by fitting the droplet profile using the Young–Laplace equation. For each MP type, three independently prepared pellets were analyzed. Five droplets were deposited at different, nonoverlapping locations on each pellet to account for surface heterogeneity, resulting in a total of 15 measurements per sample. Contact angles were recorded 30 s after droplet deposition, as no visible droplet spreading or absorption into the pellet was observed within the measurement time. The reported values therefore represent the apparent contact angle of compacted MP particle beds rather than the intrinsic contact angle of the polymer surface.

### 2.3 Aging Experiment

Biotic aging of the MPs was conducted according to our previously developed protocol^6,7,31^ in natural streamwater under controlled laboratory conditions. Around 15 L of streamwater (stream Glinščica, Ljubljana, Slovenia) was collected using grab sampling from the middle of the stream’s depth and transported to the laboratory. Stream water was used without filtration to preserve the natural microbial community. Despite the potential presence of environmental MPs in the water, the high concentration of PS and PP used (5 g/L) ensured that biofilm formation occurred on the intended particles. FTIR and microscopic analyses confirmed that the analyzed particles were exclusively PS or PP, with no evidence of contamination in the examined samples. Before starting the biotic aging experiment, the initial MP sterility was verified. Briefly, 100 mg of MPs were incubated in 5 mL of Luria–Bertani broth, a nutrient-rich medium commonly used for bacterial cultivation, for 5 days at 37 °C. No microbial growth was detected based on spectrophotometric optical density measurements (GeneQuant 1300 spectrophotometer, GE Biochrom), confirming the absence of preexisting contamination. The MPs (1 g) were placed in 250 mL Erlenmeyer flasks, containing 200 mL of streamwater. Four replicates for each MPs sample were prepared, resulting in a total of 16 Erlenmeyer flasks per sample. To facilitate gas exchange, each flask was sealed with a cotton plug. The flasks were incubated on a rotary shaker at 160 rpm and maintained at room temperature (22 ± 2 °C) with a 16-h light and 8-h dark cycle, under a light intensity of 1200–1300 lx. The samples were exposed to standard laboratory LED lighting without additional ultraviolet radiation. However, the experimental setup was also exposed to indirect natural daylight through laboratory windows, and LED illumination was used to ensure stable and continuous light conditions throughout the incubation, minimizing variability due to fluctuating natural light availability. Weekly, MPs were filtered using cellulose filters (7–12 μm pore size, Macherey-Nagel, Germany) and transferred to freshly collected streamwater. The aging proceeded over a period of 16 weeks. Stream water samples were stored at −18 ± 2 °C. After the end of the experiment, the remaining MPs were filtered and analyzed.

### 2.4 Characterization of Biofilm

#### 2.4.1 Amount of Biofilm

The mass of biofilm on the MPs was determined by calculating the difference in weight between the dried MPs with biofilm and the dried MPs after removal of the biofilm by Fenton oxidation. Three replicates, each containing approximately 40 mg of dried MPs with biofilm, were weighed and treated with 1 mL Fe^2+^ solution (15 g/L FeSO_4_·7H_2_O and 6 mL/L H_2_SO_4_ (97% *v/v*)) together with 1 mL 30% (*w/w*) H_2_O_2_). Biofilm digestion was carried out at room temperature (22 ± 2 °C) for 24 h. After digestion, the samples were filtered using nitrocellulose membrane filters (0.45 μm pore size, Sartorius, Germany). The filters were then dried at room temperature (22 ± 2 °C) until a constant mass was achieved and particles with the removed biofilm were reweighed. The amount of biofilm present on the MPs was calculated by subtracting the final weight of the MPs with removed biofilm from the initial weight (dried MPs with biofilm) as described in ref 7.

#### 2.4.2 Chlorophyll *a* Content

Chlorophyll *a* content in the biofilm was determined by homogenizing approximately 40 mg of MPs with biofilm (three replicates) in a mortar with 1.8 mL of cold 95% (*v/v*) ethanol (−18 ± 2 °C). The supernatant was then measured at 664 and 648 nm using a BioTek Synergy LX multimode reader (Agilent Technologies, USA). The chlorophyll *a* concentration was expressed as mg of chlorophyll *a* per gram of biofilm and calculated according to ref 32.

#### 2.4.3 Extracellular Polymeric Substances

The content of extracellular polymeric substances (EPS) within the biofilm was determined as described in ref 7. Approximately 40 mg of dried MPs with biofilm (three replicates) was treated with 3 mL of deionized water and incubated at 80 ± 1 °C for 30 min. After cooling to room temperature, the samples were filtered through cellulose acetate syringe filters (0.45 μm pore size, LLG Labware, Germany). 0.5 mL of 6% (*w/v*) phenol and 2.5 mL of 97% (*v/v*) H_2_SO_4_ were added to 1 mL of the filtrate. The absorbance was measured at 490 nm using a BioTek Synergy LX multimode reader (Agilent Technologies, USA) after cooling to room temperature. To calculate the concentration of EPS, a calibration curve was prepared using d-(+)-glucose and EPS concentration was expressed as mg EPS per gram of biofilm.

#### 2.4.4 Microbiological Analyses of Biofilm

Molecular analyses of the biofilm bacterial community were performed by using shotgun sequencing. Shotgun metagenomic sequencing was performed to characterize the bacterial, archaeal, and fungal communities of biofilms on MPs, with particular emphasis on taxonomic composition and genes involved in plastic degradation and biofilm formation, while chlorophyll *a* measurements were used as a proxy for total phototrophic biomass, potentially including both prokaryotic and eukaryotic phototrophs.

MP samples, 0.1 g each, were placed in 1.5 mL tubes with 200 μL of nuclease-free water, and the tubes were then sonicated at 40 kHz for two cycles of 20 min to detach biofilms and microbial cells from the surface of the plastics. Genomic DNA was then isolated using the FastDNA SPIN Kit for soil, with a modified protocol. After sonication, the mixture of plastic and water was transferred to the Lysing Matrix E tubes (provided in the kit), and 978 μL of Sodium Phosphate Buffer and 122 μL of MT Buffer (both provided in the kit) were added into the Lysing matrix. The following steps followed the standard procedure suggested by the manufacturer.

Shotgun metagenomic analyses were carried out to characterize the functional potential of the biofilm-associated microbial communities with particular attention to taxonomical identification and genes involved in plastic degradation and biofilm formation.

Metagenomic libraries were prepared using the TruSeq DNA PCR-Free kit (Illumina Inc., San Diego, CA, USA), and sequencing was performed by Novogene UK (Cambridge, UK) on the Illumina NovaSeq 6000 platform, generating 250 bp paired-end reads. Sequencing depth ranged from 68.7 to 92.6 million raw paired-end reads per sample (10.3–13.9 Gb), with high overall sequence quality across all libraries (effective read percentage: 99.66–99.73%; error rate: 0.01%; Q20 > 99%). Quality control of the raw sequences was conducted using FastQC (v0.11.9),^33^ while adapter and quality trimming were carried out with fastp (v v1.1.0).^34^ Reads shorter than 50 bp or with a mean Phred quality score below 30 were discarded, because the downstream analyses were based on direct read classification and functional annotation rather than metagenome assembly, sequencing depth and read-quality statistics were used to assess data set robustness.

Paired-end metagenomic reads for each sample were used to assess the taxonomical composition of the samples. All classifications used a local Kraken2 (v2.1.5)^35^ database built from the Standard microbial libraries (Bacteria, Archaea, and Fungi).^36^

The identification of genes putatively involved in plastic degradation was performed by searching the filtered metagenomic reads against a DIAMOND database (v2.1.17)^37^ built from curated sequences obtained from PlasticDB.^38^ To increase annotation reliability, searches were performed in blastx mode using stringent criteria: a minimum of 40% amino acid identity, an alignment length ≥ 50 residues, and an e-value ≤ 1 × 10^–20^. Only the best-scoring alignment for each read was retained, and all detected functions were therefore reported as putative plastic-degrading genes.

Each hit was assigned to its corresponding enzyme class (e.g., PETase, cutinase, laccase, and peroxidase) using a mapping file linking PlasticDB accessions to functional categories. The number of hits per enzyme class was summarized for each sample and normalized to the total number of reads to obtain the relative abundances. These values were used to visualize the distribution and diversity of plastic-degrading genes across the samples.

#### 2.4.5 Density

The particle density was determined with a pycnometer according to ref 39 and as described in ref 7. Briefly, the empty pycnometer was first weighed and then about 40 mg of dried MPs (pristine or aged with biofilm) were added to the pycnometer, and the total mass was recorded. Afterward, ethanol (96%) was added to the pycnometer and reweighed. The pycnometer containing only ethanol was also weighed at the end, and the density was calculated according to eq 1:

1 $$\rho =\frac{m_{2}-m_{1}}{m_{2}-m_{1}-m_{3}+m_{4}}\times \rho_{ET}$$

where ρ is the density of the pristine or aged MPs (g/cm^3^), *m*_1_ is the mass of the empty pycnometer (g), *m*_2_ is the mass of the pycnometer and MPs (g), *m*_3_ is the mass of the pycnometer with MPs and ethanol (g), *m*_4_ is the mass of the pycnometer and ethanol (g), and ρ_*ET*_ is the density of ethanol (g/cm^3^). The density of ethanol was determined based on the density of deionized water.^39^ The procedure was repeated for each sample of MPs using two different masses of the sample (m_2_). For each mass, five measurements of m_3_ were recorded to ensure consistency and accuracy. Repetition was necessary due to the rapid evaporation of ethanol during measurements, which could affect the results.

#### 2.4.6 Morphology

A field-emission scanning electron microscope (FE-SEM; Apreo 2 Thermo Fisher, USA) was used to evaluate changes in particle surface morphology. MPs were coated with a 10 nm layer of Au/Pd prior to the analysis. Microscopy was performed at an accelerating voltage of 2 kV by using an Everhart-Thornley detector. Changes in the particle shape were also observed under an optical microscope (Motic SMY-171, Motic, China) equipped with a digital camera (Moticam S3, Motic, China).

#### 2.4.7 Chemical Composition

Fourier-transform infrared (FTIR) analysis was used to confirm the chemical identity of the MP samples and to verify the formation of the biofilm by analyzing the functional groups. Analyses were performed on MPs with the biofilm intact, without prior cleaning, to capture chemical modifications associated with microbial colonization and EPS accumulation. Accordingly, FTIR spectra represent the combined signal of the MP–biofilm system (i.e., polymer surface plus attached microbial biomass and extracellular polymeric substances) rather than intrinsic chemical properties of the pristine polymer alone. FTIR spectra were acquired using a Nicolet iN10MX microscope (Thermo Fisher, USA) equipped with a cooled mercury cadmium telluride (MCT) detector and operating in transmission mode. For each sample, MPs were deposited on a BaF_2_ window and spectra were recorded with a nominal spectral resolution of 8 cm^–1^ in the range 4000–650 cm^–1^. To increase the signal-to-noise ratio, 64 scans per sample were coadded without changing the position of the sample between each scan (total acquisition time: 12 s, including dead time). OMNIC Picta software (Thermo Fisher, USA) was used for all spectra manipulations.

#### 2.4.8 Crystallinity

Differential scanning calorimetry with a DSC1 module (Mettler Toledo, Switzerland) in combination with a TC100 MT intracooler was used to characterize the melting and crystallization phenomena of a sample. Samples were placed in 40 μL aluminum crucibles and tightly sealed with lids. The weighed masses of the samples amounted to approximately 3 mg; each sample was measured in four replicates. The temperature program of the measurement, which was carried out under a constant nitrogen flow (Messer 5.0) of 30 mL/min, consisted of several steps. After an initial 3 min isothermal conditioning at 25 °C, the sample was dynamically heated to 80 °C, and after 2 isothermal minutes at this temperature, the sample was heated to 180 °C. It was then cooled from the molten state to 0 °C and reheated to 180 °C. The dynamic parts of the measurement were performed at a rate of 10 °C/min. The first heating removed any thermal history of the samples, and the second heating run was analyzed to determine the melting temperatures and enthalpy of melting (fusion) to determine the degree of crystallinity by peak area integration of each sample.

Equation 2 was used to determine the degree of crystallization, *X*_c_ (%), using

2 $$X_{C}=\frac{\Delta H_{m2}}{\Delta H_{m}^{{}^{\circ}}}$$

where Δ*H*_m2_ is the melting enthalpy from the second heating run (J/g) and Δ*H*°_m_ is the melting enthalpy of a 100% crystalline material (J/g). For PP, Δ*H*°_m_ is given as 207 J/g.^40^

### 2.5 Data Analysis

The data on MP biofilm mass, chlorophyll *a* content, EPS, density, and crystallinity analysis are expressed as the mean ± standard deviation. Statistical comparisons were made between the two milling methods for each plastic type. Normality and homogeneity of variances were tested using the Shapiro-Wilk test and Levene’s test, respectively. For biofilm mass, chlorophyll *a* content, EPS, and crystallinity, the data were normally distributed and homogeneous, so a Student’s *t* test was applied. For density analysis, the data were not normally distributed, and the Mann–Whitney U test was used. For biofilm parameters (biofilm mass, chlorophyll *a*, and EPS), a two-way analysis of variance (ANOVA) was performed to evaluate the effects of polymer type (PS vs PP), milling method (ultracentrifugal mill vs mixer mill), and their interaction (polymer × milling). Differences were considered statistically significant if *p* < 0.05. Additionally, principal component analysis (PCA) was performed on the biofilm parameters. This multivariate statistical method simplifies complex data sets by transforming correlated variables into a smaller number of uncorrelated components, thus highlighting patterns and groupings in the data.^41^ All analyses were performed using OriginPro 2024 software (OriginLab Corp., USA).

## 3 Results

### 3.1 Test Microplastic Characterization

To better understand the origin of the differences observed during biotic aging, we characterized the physicochemical properties of pristine MPs produced by the two fragmentation techniques prior to biofilm formation. Since microbial attachment is known to be influenced by the initial surface characteristics of MPs, particle size distribution, particle morphology, and apparent wettability were evaluated before exposure to streamwater. The detailed characterization is reported in the Supporting Information, while the main findings relevant to biofilm development are reported below.

Particle size analysis confirmed that both fragmentation methods produced MPs with comparable upper size limits (<500 μm), although distinct particle size distributions were observed depending on the milling technique (Figure S1). In addition, quantitative image analysis revealed significant differences in particle morphology (Figures S2–S4). MPs produced by mixer milling exhibited significantly lower convexity values than ultracentrifugally milled particles for both PS and PP, indicating a higher degree of particle edge irregularity. In contrast, no significant differences in solidity were observed, suggesting that fragmentation mainly affected the local complexity of the particle outline without substantially altering the overall particle shape.

Differences in the particle morphology were accompanied by changes in apparent wettability. Contact angle measurements performed on compacted MP pellets showed that mixer-milled MPs exhibited significantly higher apparent contact angles than ultracentrifugally milled particles for both polymers (Figure S5), indicating that the fragmentation technique also modifies the interfacial properties of the resulting particles. The increase in the apparent contact angle was particularly pronounced for PS, whereas a smaller but still significant increase was observed for PP.

### 3.2 Biofilm Characterization

The formation of biofilms on the surface of PS and PP MPs, prepared by two different milling techniques (ultracentrifugal mill and mixer mill), was analyzed by evaluating the amount of biofilm, EPS, chlorophyll *a* content, and microbial composition.

PS MPs, prepared with the ultracentrifugal mill, had a greater amount of biofilm compared to those prepared with the mixer mill (Figure 2). The amount of biofilm, as well as the concentrations of EPS and chlorophyll *a*, was higher on MPs prepared with the ultracentrifugal mill. Statistically significant differences in both EPS and chlorophyll *a* contents were observed between the milling techniques, with the ultracentrifugal mill resulting in higher values of these parameters.

**Figure 2:**
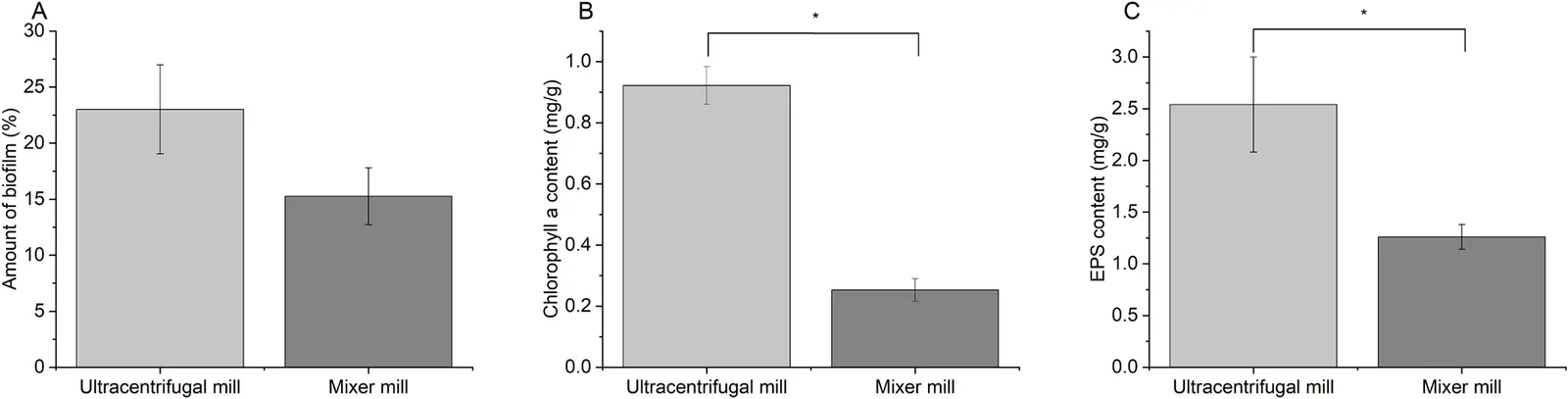
Comparison of biofilm parameters between polystyrene microplastics prepared with the ultracentrifugal mill and the mixer mill: A) amount of biofilm, B) extracellular polymeric substances (EPS), and C) chlorophyll *a* contents. The asterisk indicates a significant difference between the different milling techniques for each parameter.

In contrast to the PS results, the amount of biofilm on PP MPs was significantly lower for those produced by the ultracentrifugal mill compared to those prepared with the mixer mill (Figure 3). For both EPS and chlorophyll *a* contents, lower amounts were observed in the mixer mill samples, although the difference was only statistically significant for the chlorophyll *a* content.

**Figure 3:**
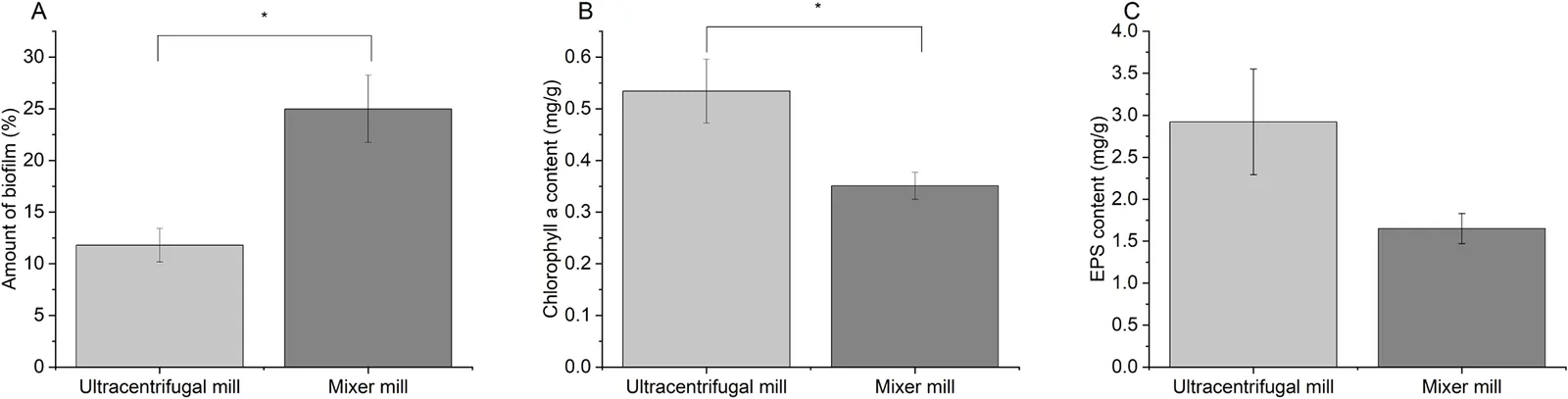
Comparison of biofilm parameters between polypropylene microplastics prepared with the ultracentrifugal mill and the mixer mill: A) amount of biofilm, B) extracellular polymeric substances (EPS), and C) chlorophyll *a* contents. The asterisk indicates a significant difference between the different milling techniques for each parameter.

Two-way ANOVA revealed parameter-specific effects of polymer type and milling method. For biofilm amount, a significant Polymer × Milling interaction (*F*(1, 8) = 24.70, *p* = 0.001, η^2^p = 0.76) was observed, while main effects were not significant (polymer: *F*(1, 8) = 0.13, *p* = 0.73, η^2^p = 0.02; milling: *F*(1, 8) = 1.66, *p* = 0.23, η^2^p = 0.17), indicating polymer-dependent milling responses. For chlorophyll *a*, significant main effects of both polymer type (*F*(1, 8) = 17.65, *p* = 0.003, η^2^p = 0.69) and milling method (*F*(1, 8) = 152.78, *p* < 0.0001, η^2^p = 0.95) were detected, together with a significant interaction (*F*(1, 8) = 49.65, *p* < 0.001, η^2^p = 0.86), demonstrating that phototrophic activity is strongly influenced by both factors and their interplay. For EPS production, only the milling method showed a significant main effect (*F*(1, 8) = 20.16, *p* = 0.002, η^2^p = 0.72), with no significant polymer (*F*(1, 8) = 1.87, *p* = 0.208, η^2^p = 0.19) or interaction effects (*F*(1, 8) ≈ 0.00, *p* = 0.98, η^2^ *p* < 0.001), suggesting a consistent and strong influence of fragmentation technique across materials.

The PCA of the biofilm parameters yielded a two-component model accounting for 91.44% of the total variance (52.39% for Principal Component 1 (PC1) and 39.05% for Principal Component 2 (PC2)). The biplot reveals four distinct clusters corresponding to the intersection of material type (PP and PS) and milling technique (ultracentrifugal mill and mixer mill) (Figure 4). PC1, which explains the majority of the variance, primarily discriminates samples based on the milling technique. Samples processed via the mixer mill (represented by circles) are situated exclusively in the negative PC1 space, while samples processed via the ultracentrifugal mill (represented by squares) are clustered in the positive PC1 space. Principal Component 2 (PC2) further separates the samples by material type, although the vertical distribution is dependent on the milling method. The loading vectors indicate that chlorophyll *a* and EPS are positively correlated with PC1, pointing toward the ultracentrifugal milled PS cluster. The “Amount of biofilm” vector points toward the upper-left quadrant, aligning most closely with the mixer-milled PP samples.

**Figure 4:**
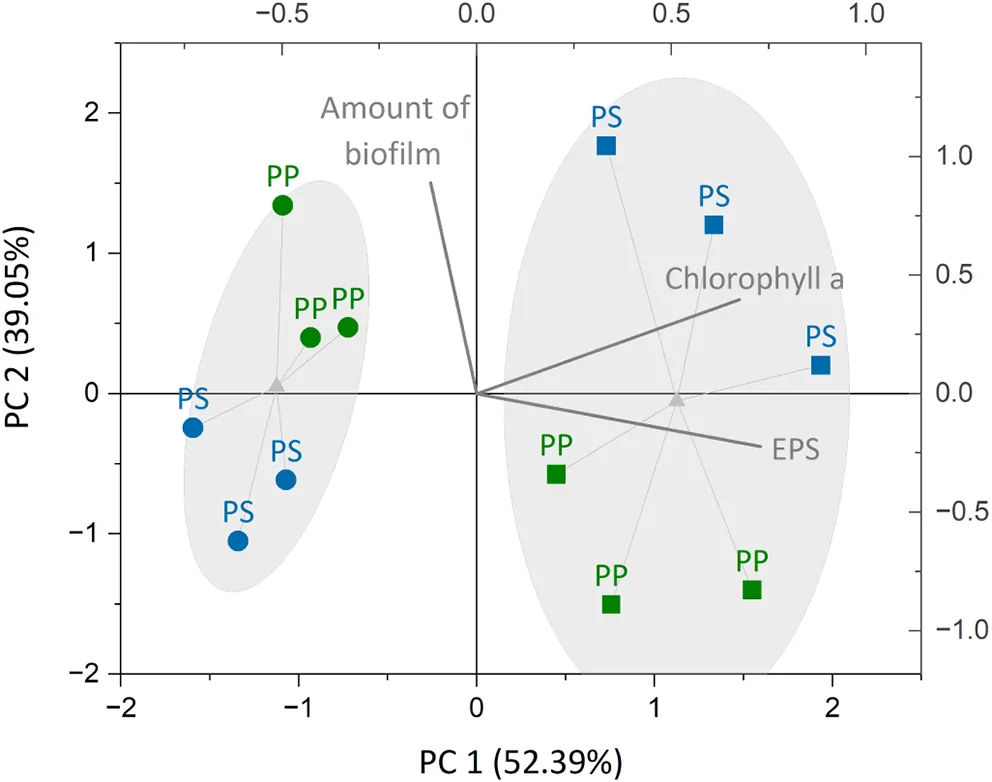
Principal component analysis biplot of microplastic characteristics based on polymer type and milling technique. The score plot illustrates the distribution of polystyrene (PS; blue) and polypropylene (PP; green) samples processed via the mixer mill (circles) and the ultracentrifugal mill (squares). The first two principal components (PC1 and PC2) account for 52.39% and 39.05% of the total variance, respectively. Gray vectors represent the loading variables: amount of biofilm, chlorophyll *a* content, and extracellular polymeric substances (EPS) content. Centroids and spider lines represent the mean and dispersion of the milling technique groups.

#### 3.2.1 Microbial Composition of Biofilm

Taxonomic profiling of the metagenomic reads revealed a broadly similar bacterial community composition across all MP samples. The genus *Brevibacillus* represented the most abundant taxon (Figure 5) in all samples, indicating a dominant role in MP colonization under the conditions tested. While the same major genera were detected in all treatments, differences in their relative abundances were observed. *Brevibacillus* appeared elevated in samples processed with the ultracentrifugal mill for both PS and PP polymers, and *Paenibacillus* also appeared relatively stable and consistently represented across all conditions, without a strong indication of polymer- or fragmentation-specific enrichment. Phototrophic bacteria were identified within the core microbiota, notably *Bradyrhizobium*, known for its aerobic anoxygenic phototrophic activity.^42^ However, the increase observed in the chlorophyll *a* concentration was not accompanied by a corresponding change in the relative abundance of this genus. Conversely, some genera exhibited different abundances that were associated with the fragmentation method. In particular, *Clostridium* displayed higher abundance in samples obtained with the ultracentrifugal mill compared to their counterparts obtained with the mixer mill, whereas *Priestia* appeared more abundant in samples fragmented with the latter. The data indicate that the mechanical fragmentation process adopted to produce test MPs does not alter the overall community membership but drives a redistribution in the proportional representation of key genera, most notably *Brevibacillus*, *Clostridium*, and *Priestia*.

**Figure 5:**
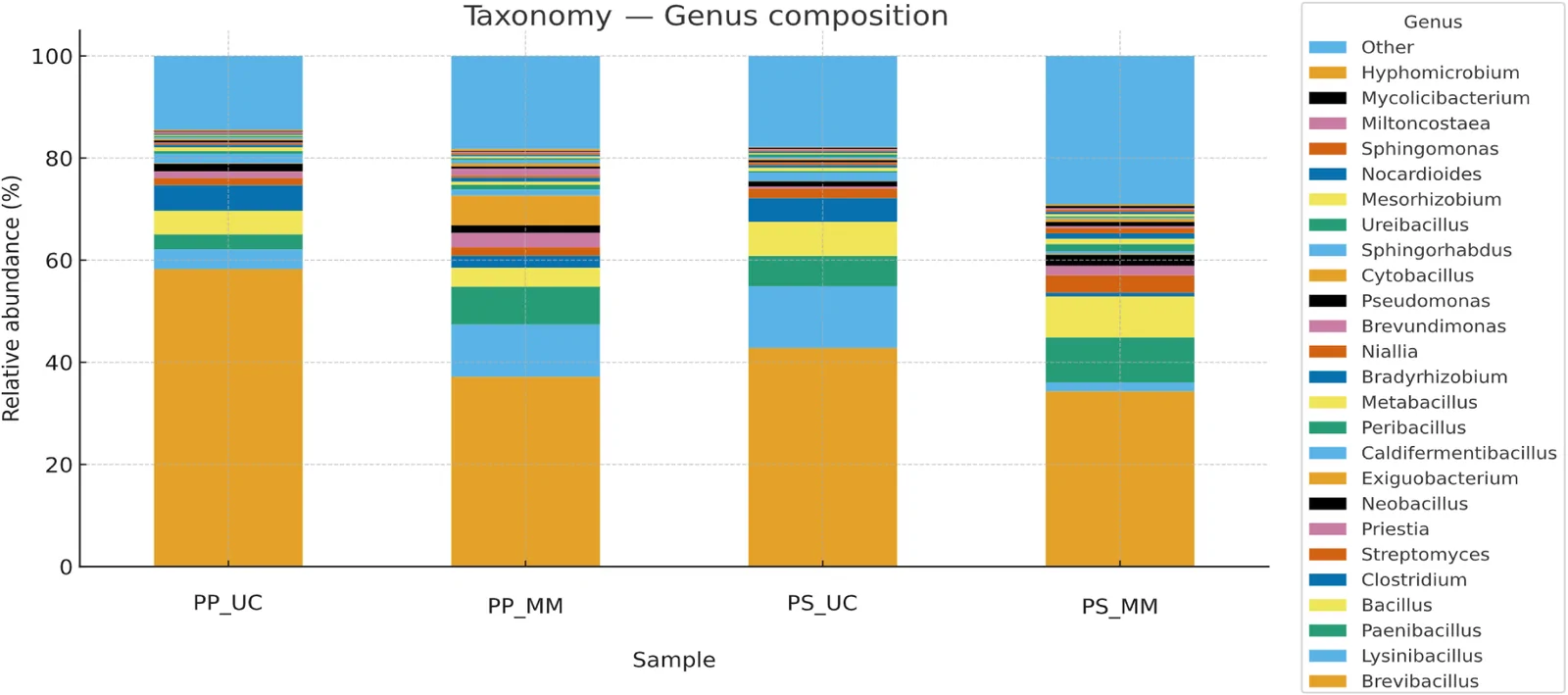
Relative abundance of the top 25 bacterial genera detected in metagenomic data sets from biofilms on polypropylene (PP) and polystyrene (PS) microplastics produced by ultracentrifugal (UC) or mixer milling (MM).

#### 3.2.2 Functional Screening for Plastic-Degradation Genes

Functional annotation of high-quality metagenomic reads against the PlasticDB enzyme database identified multiple protein families putatively involved in polymer degradation (Figure 6B). Detected enzyme classes included oxidative enzymes such as laccases and peroxidases and hydrolytic enzymes such as cutinases, monooxygenases, and PETases. Laccases and peroxidases represented the predominant enzyme categories across all data sets, whereas PETase-related hits were detected at low but consistent frequencies in every sample. Normalization by total read count revealed differences in the abundance of plastic-degrading gene hits that were dependent on both polymer type and fragmentation method (Figure 6A). PS samples produced with the ultracentrifugal mill (PS-UC) showed a higher relative abundance of putative plastic-degrading enzymes compared to the corresponding mixer-milled PS samples (PS-MM). Conversely, in PP samples, the mixer-milled samples (PP-MM) exhibited a greater abundance of enzyme hits than did PP-UC. These trends match the previously observed differences in biofilm accumulation on the same materials, with higher enzyme representation under the conditions that also displayed increased biofilm formation. Variations in enzyme class representation were also visible between treatments, although oxidative enzymes (e.g., laccases and peroxidases) remained among the most represented categories in all of the samples.

**Figure 6:**
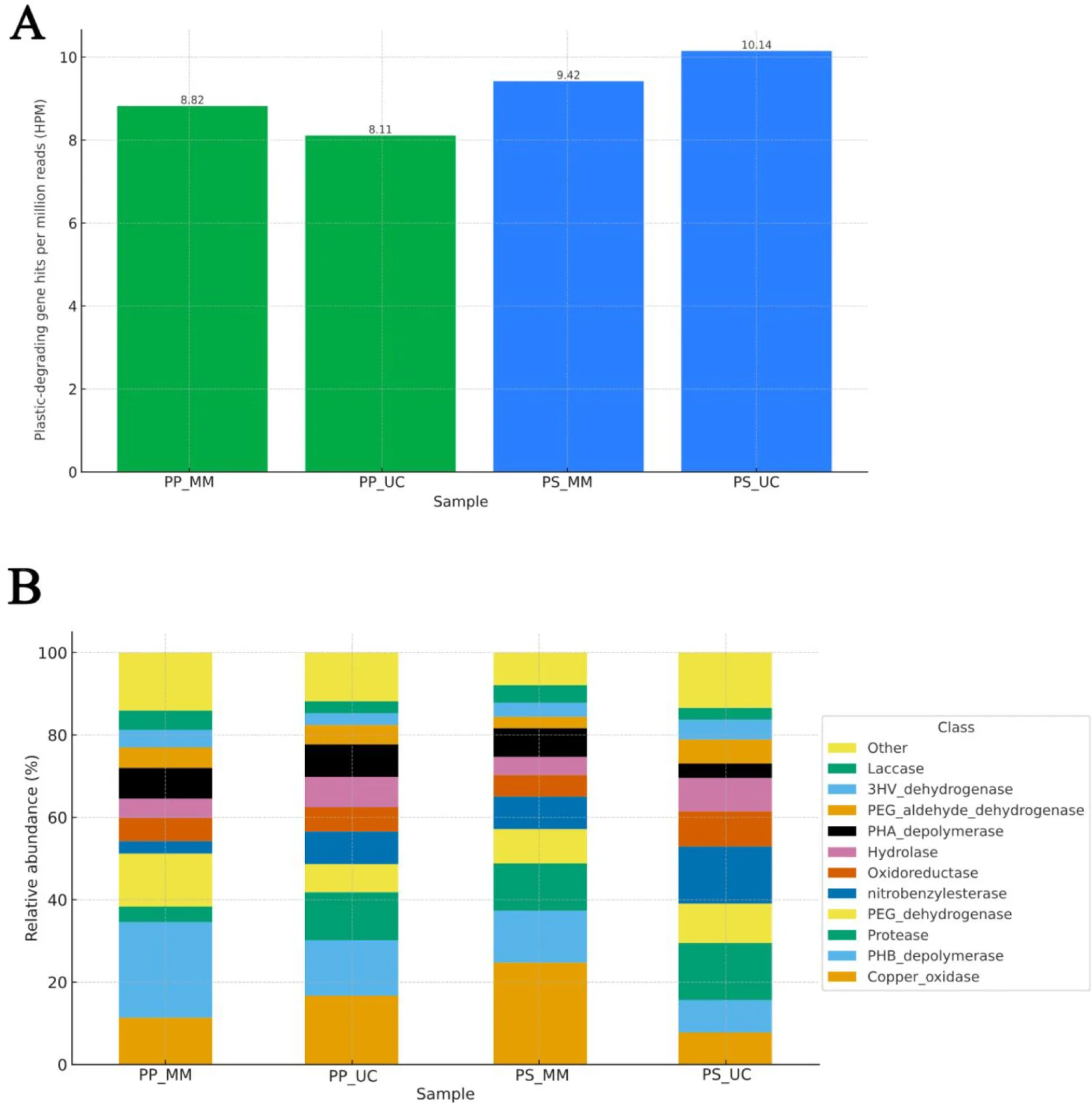
A) Normalized biofilm gene hit counts per sample (expressed as hits per million reads) and B) distribution of plastic-degrading genes identified across samples (illustrating the functional potential for polymer breakdown) on polypropylene (PP) and polystyrene (PS) microplastics produced by ultracentrifugal (UC) or mixer milling (MM).

### 3.3 Analysis of Microplastics

The MPs were analyzed for changes in density, morphology, chemical composition, and crystallinity before and after aging regarding the influence of the two milling techniques.

#### 3.3.1 Density

When comparing the densities of pristine PS and PP MPs, no significant differences were observed between the two milling techniques (Figure 7). However, for both aged PS and PP MPs, there were statistically significant differences in the density between aged samples, prepared with an ultracentrifugal mill and a mixer mill (Figure 7).

**Figure 7:**
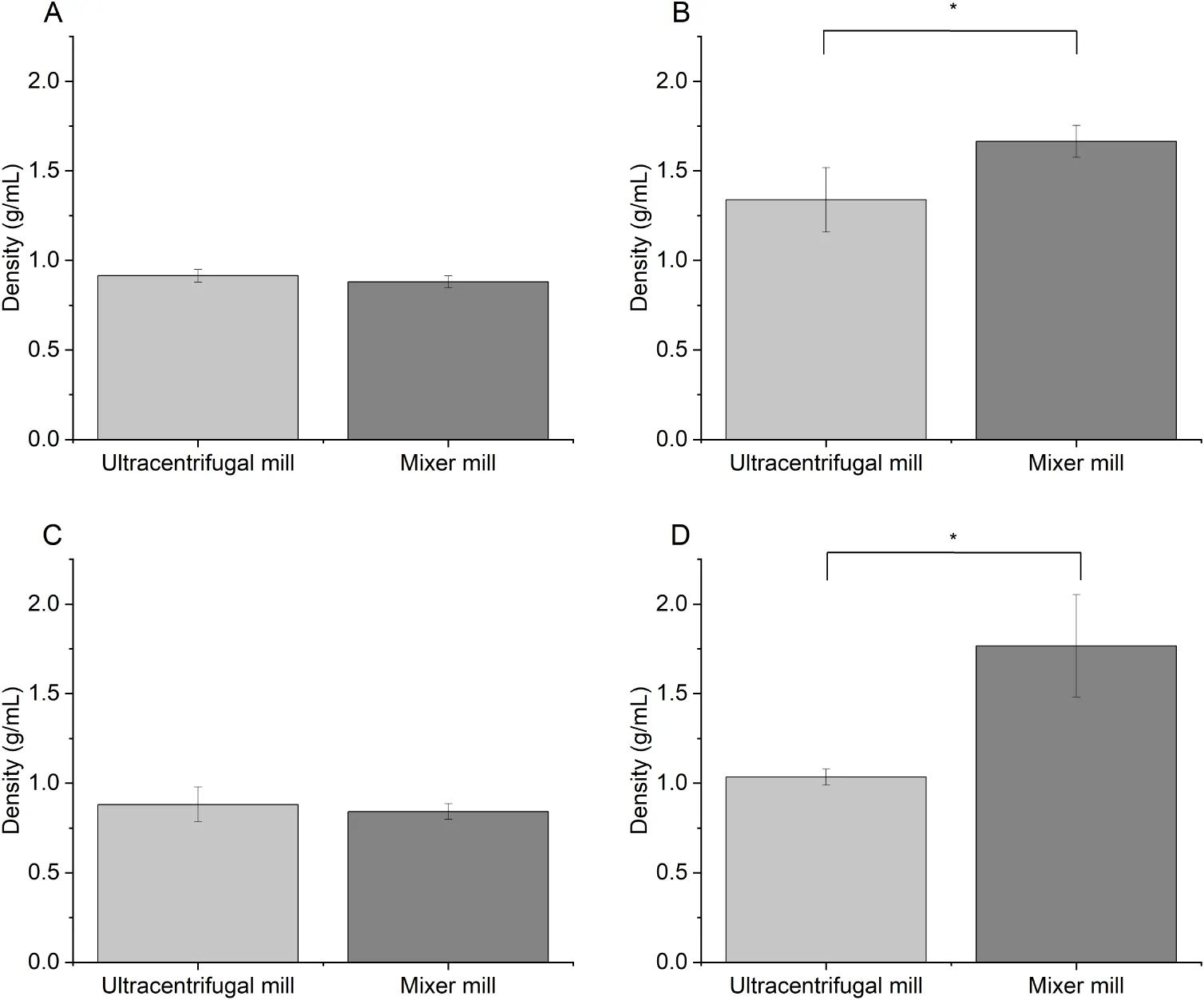
Comparison of density between microplastics prepared with the ultracentrifugal mill and the mixer mill before and after aging: A) pristine polystyrene, B) aged polystyrene, C) pristine polypropylene, and D) aged polypropylene. The asterisk indicates a statistically significant difference in density between the different milling techniques.

#### 3.3.2 Morphology

Morphological analysis is shown in Figure 8, which presents optical microscope and FE-SEM images of both types of MPs, alongside a side-by-side comparison of samples prepared using two different milling techniques. For both PS and PP, it is clear that particles processed with the mixer mill are less bulky and more fragmented. In contrast, particles processed with the ultracentrifugal mill exhibit cleaner and sharper edges. Additionally, FE-SEM images of aged PP show variations in microbial colonization, with distinct types of microorganisms present depending on the milling technique used.

**Figure 8:**
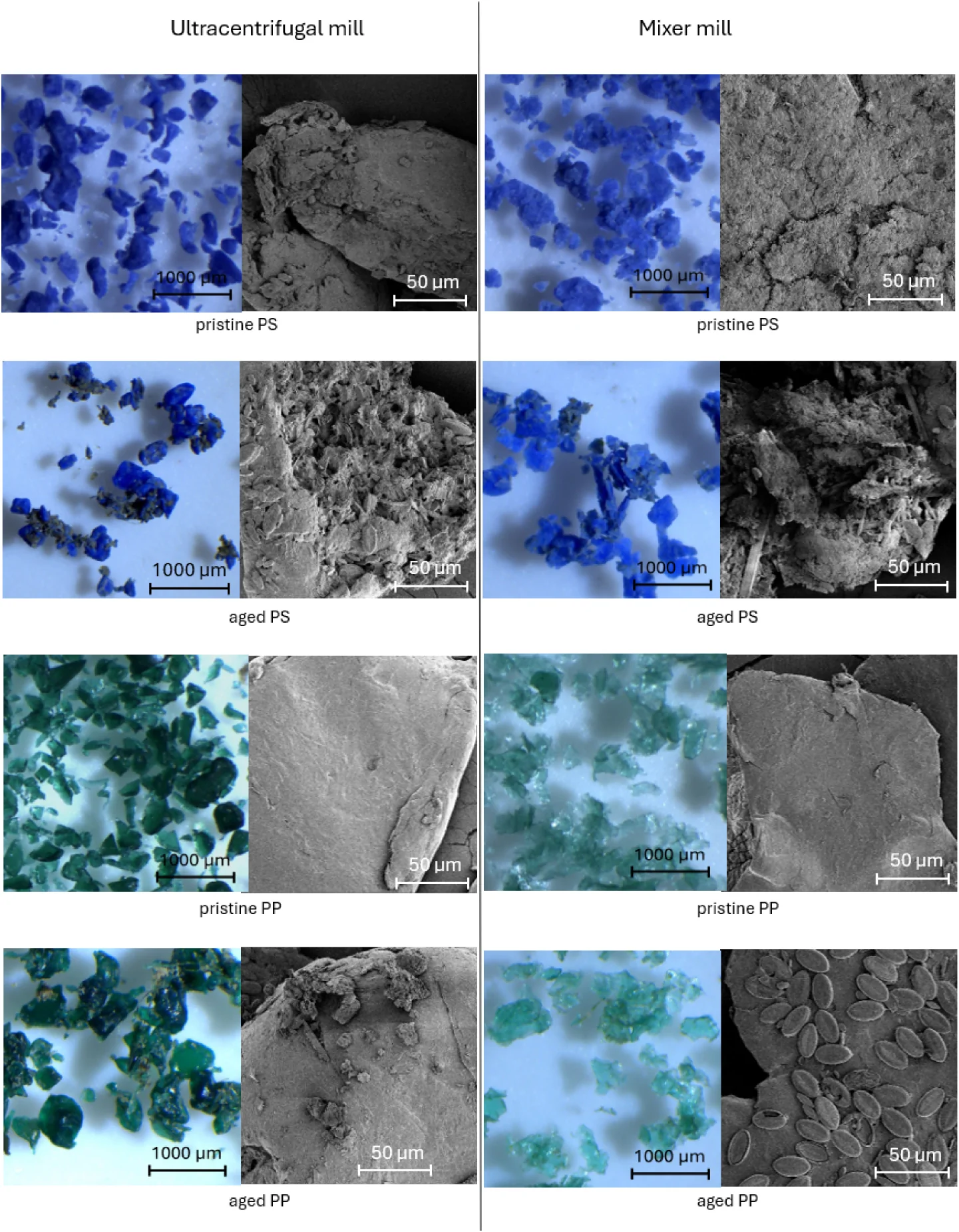
Analysis of morphology with an optical microscope and a field emission scanning electron microscope: polypropylene (PP) and polystyrene (PS) microplastics, prepared with the ultracentrifugal mill and the mixer mill, before and after aging.

#### 3.3.3 Chemical Composition

The FTIR spectra of all plastics showed changes that can be attributed to the formation of a biofilm on the surface of the MPs and reveal new functional groups that can be assigned to biomolecules such as lipids, proteins, nucleic acids, and carbohydrates (Figure 9). In the spectra of aged PP MPs, the following changes can be observed: a broadening of the spectral region between 3570 and 3200 cm^–1^ due to O–H stretching; double peaks at 3344 and 3405 cm^–1^ due to N–H stretching of aliphatic amine I; peaks around 1790 cm^–1^ and 1735 cm^–1^ due to acyl and ester carbonyl groups; a peak at around 1635 cm^–1^ due to the absorption of amide I and a peak at about 1530 cm^–1^ due to the adsorption of amide II; an increase in the intensity of the peak at about 1215 cm^–1^ probably due to the C–N symmetry vibration of amide III; an increase in the intensity of the peaks at 1100 cm^–1^ and 1050 cm^–1^ due to the C–O stretching of primary and secondary alcohols. In the spectra of aged PS MPs, the spectral changes are more difficult to observe due to the complexity of the PS spectrum. However, it is possible to highlight the following: a broadening of the spectral region between 3570 and 3200 cm^–1^ due to O–H stretching; a broadening and increase in intensity of the peak at 1650 cm^–1^ of amide I absorption; a broadening of the methyl and methylene C–H absorption region between 1480 and 1340 cm^–1^.

**Figure 9:**
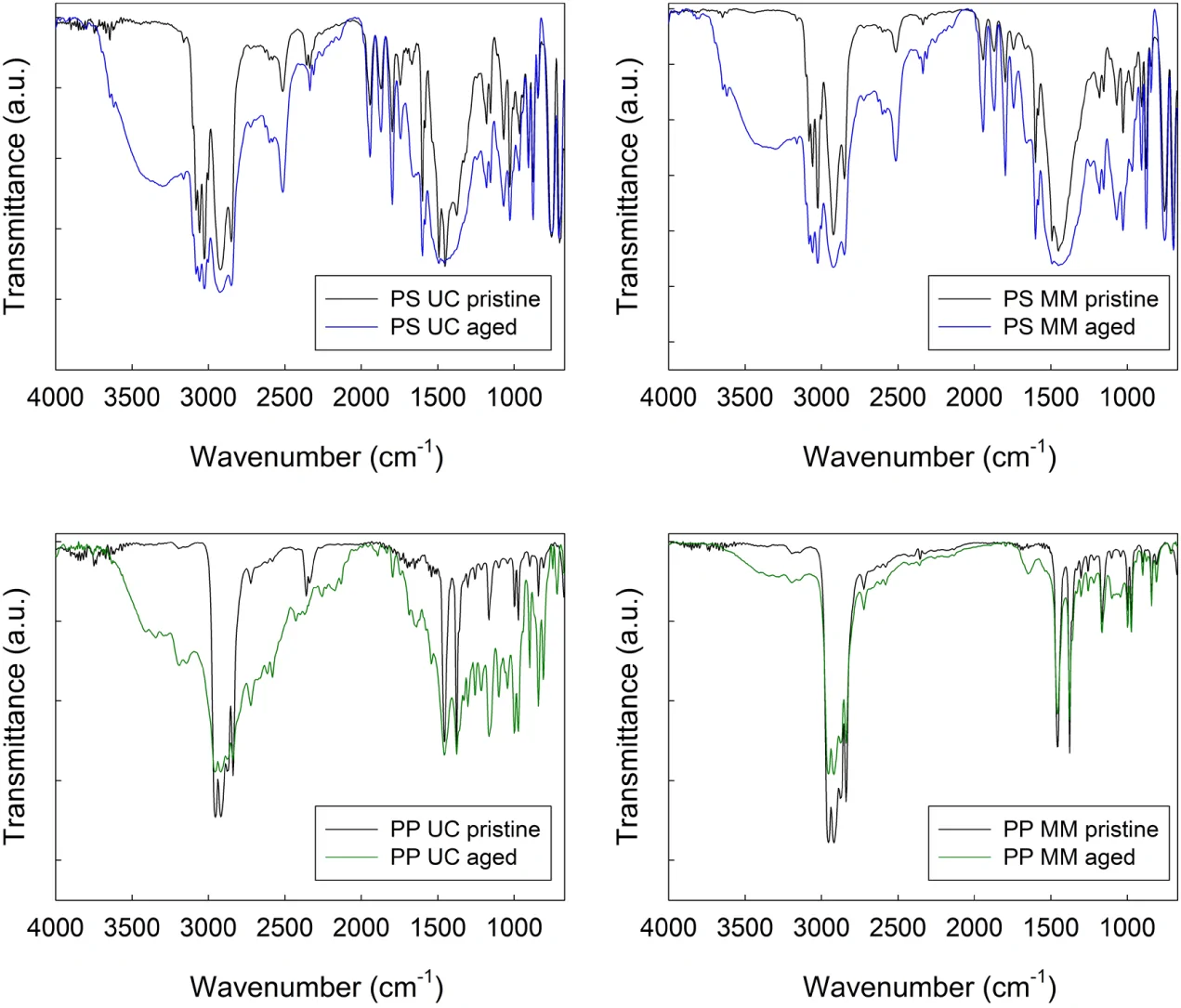
Fourier-transform infrared spectroscopy analysis of pristine and aged polystyrene (PS) and polypropylene (PP) microplastics prepared with the ultracentrifugal mill and the mixer mill.

#### 3.3.4 Crystallinity

All tested PS MPs were in amorphous form (atactic PS), and therefore, showed no melting up to 300 °C. In the temperature range above 80 °C, the change in heat capacity indicated softening, which was determined as a glass transition with a midpoint temperature of about 90 °C. Therefore, changes in the internal structure of PS following different milling techniques could not be evaluated. In contrast, for PP MPs, the crystallinity of pristine PP showed no significant differences between MPs prepared by the ultracentrifugal and mixer mill (Figure 10). However, in the case of the aged PP samples, a significant difference in crystallinity was observed. Specifically, PP MPs produced with the ultracentrifugal mill exhibited higher crystallinity compared to those produced with the mixer mill.

**Figure 10:**
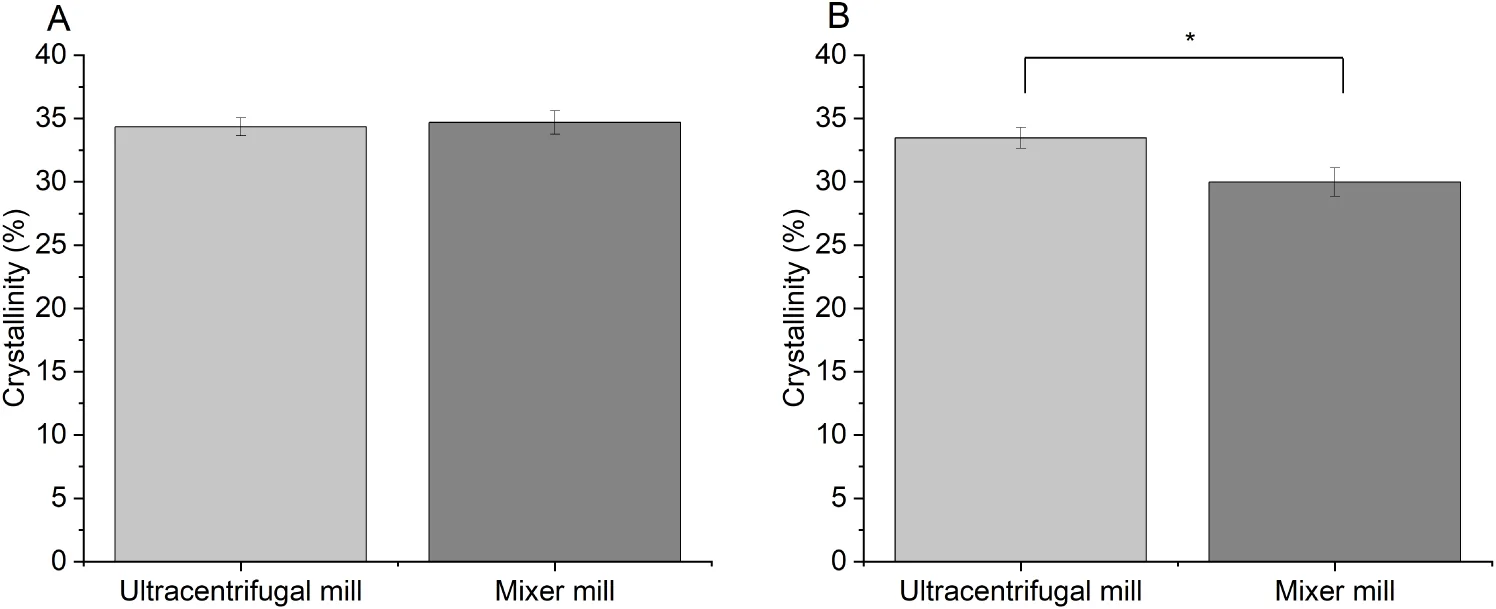
Comparison of crystallinity between polypropylene microplastics prepared with the ultracentrifugal mill and the mixer mill before and after aging: A) pristine polypropylene and B) aged polypropylene. The asterisk indicates a significant difference in density between the different milling techniques.

## 4 Discussion

The results of this study provide important insights into the effects of different fragmentation techniques on the biotic aging of MPs, particularly with regard to biofilm formation, microbial composition, and changes in MP properties. The comparison between ultracentrifugal milling and mixer milling reveals significant differences in biofilm development, suggesting that the initial morphological and physicochemical properties of MPs play a crucial role in their interactions with microbial communities in aquatic environments. The two fragmentation techniques rely on different mechanical forces to simulate the natural breakdown of plastics driven by wave action and collisions with rocks. In ultracentrifugal milling, particle size reduction results from the combined action of impact and shear forces generated between a rapidly rotating rotor and a stationary ring sieve. As plastic fragments enter the system, centrifugal acceleration propels them outward at high velocity, causing repeated impacts against wedge-shaped rotor teeth and subsequent fragmentation. Additional size reduction occurs through further interactions between the rotor and the ring sieve, ultimately yielding a fine powder. Mixer milling, on the contrary, operates through impact and friction mechanisms. Plastic materials are placed in a rotating or oscillating chamber together with small stainless-steel balls, typically a few millimeters in diameter. The continuous motion causes the plastic pieces to collide with the balls and the inner walls of the chamber, progressively reducing their size. As a result of different forces acting on plastics, the obtained microparticles generally present different properties, such as size distribution, particle morphology, and surface wettability, depending on both the plastic type and the fragmentation technique.^24^ Biotic aging of MPs prepared using these two different fragmentation methods was evaluated through several parameters. First, biomass accumulation on the particle surfaces was assessed, and the results demonstrated that biofilm formation was significantly influenced by both the type of polymer and the method of fragmentation. PS fragmented via ultracentrifugal milling exhibited greater biofilm formation compared to MPs produced by mixer milling, although these differences did not reach statistical significance. In contrast, for PP, statistically significant differences were observed, with MPs fragmented by mixer milling supporting more extensive biofilm growth. This observation is clearly mirrored in the PCA biplot (Figure 4), where the “Amount of Biofilm” loading vector aligns specifically with the quadrant containing PP samples processed via mixer milling. The spatial separation along PC2 (39.05% variance) further confirms that PP and PS exhibit diverging biological affinities depending on the mechanical stress applied during fragmentation. Interestingly, contact angle measurements showed that mixer-milled MPs exhibited significantly higher apparent contact angles than ultracentrifugally milled MPs for both polymers (Figure S5A), which is opposite to the biofilm responses observed for PS and PP. Similarly, mixer-milled MPs generally exhibited lower convexity than ultracentrifugally milled MPs (Figure S3), indicating that mixer milling produced MPs with more irregular surface features that could potentially provide favorable sites for microbial attachment. This disparity suggests that surface morphology and the wettability alone does not fully account for the observed patterns; if it did, one would expect consistently higher biofilm accumulation on more irregular and wettable surfaces across both polymer types. Instead, the interplay between surface morphology and surface chemistry, such as the presence of specific functional groups introduced or altered during fragmentation, appears to be involved in mediating microbial community composition. The PCA results support this multifactorial interpretation, as the primary axis (PC1, 52.39% variance) primarily discriminates samples by milling technique, suggesting that the fragmentation method induces fundamental changes in MPs that supersede polymer identity in importance.

In addition to surface properties, particle size distribution may also contribute to the observed differences in biofilm formation. Particle size is known to influence microbial colonization by affecting the available surface area and microhabitats for attachment. The fragmentation techniques used in this study inherently generate different particle size distributions, as previously demonstrated in our earlier work,^24^ and confirmed here for the MPs produced for the present study (Supporting Information, Figure S1). Although all samples were sieved to <500 μm, the two milling techniques produced distinct particle size distributions within this size range, and this variability may still influence microbial attachment and subsequent biofilm development. Consequently, the differences observed in biofilm formation are likely driven by the combined influence of particle size distribution, particle morphology, interfacial properties, and other physicochemical modifications induced by the milling process, rather than by a single parameter alone. A limitation of this study is that the relative contributions of these parameters induced by fragmentation could not be fully disentangled, as these parameters are intrinsically interdependent in mechanically produced MPs. In addition, although results were normalized by mass, differences in effective surface area arising from fragmentation-induced morphology may still influence microbial attachment and biofilm accumulation, representing a potential source of bias in the comparison. Importantly, these differences are not experimental artifacts, but rather intrinsic consequences of the fragmentation techniques commonly used to generate MPs for laboratory studies.

This is further supported by the measurement of chlorophyll *a* content in the biofilms, which serves as an indicator of the metabolic activity of photosynthetic microorganisms and differed significantly depending on the fragmentation method. MPs produced by ultracentrifugal milling consistently showed a significantly higher chlorophyll *a* level, indicating a more active community of phototrophic microorganisms. This was accompanied by increased levels of EPS, a key component of biofilm structure and stability, suggesting an increased level of microbial metabolic activity. In the PCA biplot, the chlorophyll *a* and EPS vectors point strongly toward the positive PC1 space, specifically clustering with the ultracentrifugally milled PS samples. This tight correlation suggests that ultracentrifugal milling creates a surface environment that is specifically conducive to photosynthetic colonization. These results are consistent with our previous studies, in which biofilms on spheres with smooth surfaces had more EPS than those on fragments and films with irregular textures, likely due to a greater need for structural support.^22^ Taken together, these results highlight the multifactorial nature of biofilm formation on MPs, where both physical and chemical surface properties, shaped by polymer type and processing method, control microbial attachment, succession, and ecological function within the developing biofilm. The high cumulative variance explained by the PCA (91.44%) reinforces the conclusion that these three parameters, the amount of biofilm, chlorophyll *a* content, and EPS content, are highly sensitive and reliable indicators of the changes induced by fragmentation. Importantly, the influence of fragmentation method on biotic aging parameters was parameter-specific and, in some cases, polymer-dependent, underscoring that preparation techniques can differentially modulate biological responses depending on the material and the end point considered.

Metagenomic profiling indicates that biofilm communities on PP and PS MPs share a similar set of dominant genera with differences primarily in their relative abundances. *Brevibacillus* is consistently the most abundant genus across all conditions, in line with reports of *Brevibacillus* colonizing plastic surfaces and exhibiting biofilm-forming capacity.^43–45^ Within this broadly conserved community, some genera vary with the fragmentation methods. *Clostridium* is more abundant in MPs processed with an ultracentrifugal mill, compared with those obtained with a mixer mill, whereas *Priestia* is comparatively higher in MP produced by a mixer mill. These shifts point to a redistribution within a stable taxonomic core rather than a complete shift in community composition. *Bradyrhizobium* is also ranked among the top 25 genera across all of the treatments. Its occurrence in plastisphere communities has been reported previously, where it is listed among key taxa detected on plastic-associated biofilms.^46^ Moreover, several *Bradyrhizobium* lineages are aerobic anoxygenic phototrophs that harbor photosynthesis gene clusters and light-harvesting complexes. These features are consistent with the detection of a phototrophic signal in the biofilms, yet *Bradyrhizobium* shows little change in its relative abundance across treatments. This combination suggests that the observed increase in chlorophyll *a* under ultracentrifugal mill processing for PS, together with the lower values observed for PP under the same fragmentation technique, may reflect differences in metabolic potential or physiological state within the biofilm rather than major shifts in the *Bradyrhizobium* abundance. It should also be noted that chlorophyll *a* measurements provide a proxy for total phototrophic biomass and may reflect contributions from both prokaryotic (e.g., cyanobacteria) and eukaryotic phototrophs (e.g., algae and other microeukaryotes). Since the present study did not include targeted 18S rRNA sequencing or equivalent eukaryotic-specific analyses, the relative contribution of eukaryotic versus prokaryotic phototrophs to the observed chlorophyll *a* signal cannot be resolved. Aerobic anoxygenic phototrophs such as *Bradyrhizobium* can, in fact, adjust their metabolism between photoheterotrophic and heterotrophic modes, which provides an explanation for stable taxonomic representation alongside changes in the phototrophic signal. These results support a view in which biofilm repartition and local microenvironment, shaped by polymer type and milling, can influence their metabolic potential and ecological organization of the biofilm community.

The enzyme hit profiles were consistent with the biofilm patterns. Total plastic-degrading hits per million reads are higher in PS produced by an ultracentrifugal mill than a mixer mill and higher in PP produced by a mixer mill than an ultracentrifugal mill, mirroring the respective increases in biofilm and EPS measured for those conditions. This concordance indicates that the conditions associated with greater biofilm biomass also harbor a higher representation of genes putatively involved in plastic degradation. However, it is important to emphasize that metagenomic profiling reflects the presence and relative abundance of genetic sequences rather than their active expression or their enzymatic functionality. Therefore, the detected genes should not be interpreted as direct evidence of ongoing plastic degradation activity, which would require complementary transcriptomic, proteomic, and enzymatic validation. Differences between polymers were also evident in the abundance of detected gene hits, with PS MPs produced by an ultracentrifugal mill showing the highest overall hits and PP MPs produced by an ultracentrifugal mill showing the lowest.

The composition of enzyme classes varies with treatment, without a single uniform shift across all classes. Oxidative functions, including copper oxidases and laccases, contribute a substantial fraction in every sample, while hydrolytic categories such as depolymerases, hydrolases, and proteases are consistently present. It should be noted that oxidative enzymes such as laccases and peroxidases are widely distributed in natural aquatic environments and are primarily involved in the transformation of diverse natural organic matter.^47,48^ Given their broad substrate specificity, their high abundance in the present study should not be interpreted as specific evidence of PP or PS degradation but rather as part of the general oxidative metabolic potential within the biofilm community. The relative contributions of several classes differ between ultracentrifugal and mixer mills within a polymer and between polymers within the same milling method. For example, copper oxidase representation is elevated in PS MPs produced by a mixer mill relative to those produced by an ultracentrifugal mill, whereas PHB-depolymerase representation is higher in MPs produced by an ultracentrifugal mill. PP samples display comparatively higher proportions of dehydrogenase classes that are linked to small-molecule degradation. These differences suggest fragmentation- and polymer-associated variation in the genetic potential for oxidative and hydrolytic functions rather than a single dominant enzymatic signature.

While the difference in color between PS and PP could also influence microbial colonization or phototrophic activity, we primarily compared different milling techniques within the same material and between materials to determine whether the observed trends in biofilm formation and community structure were driven by milling rather than polymer type.

In addition to evaluating the biological parameters, the study also examined the changes in the physicochemical properties of the MPs after aging. FTIR spectroscopy confirmed the presence of functional groups associated with biofilm formation, indicating the successful microbial colonization of the aged particles. FE-SEM examination revealed morphological differences between the MPs produced by different milling methods. Both PS and PP MPs produced by mixer milling were more fragmented and had a more irregular surface than those produced by ultracentrifugal milling. The greater surface irregularity probably resulted in a larger specific surface area, especially in the case of PP MPs, which favored the adhesion of microorganisms and the development of a biofilm. This is consistent with the significantly greater amount of biofilm observed on the PP particles produced by mixer milling. This observation is consistent with previous studies reporting that rougher MP surfaces promote greater microbial attachment and biofilm development due to increased surface area and available anchoring sites.^22,49^

While no significant differences in density were observed between pristine PS and PP MPs produced by either milling technique, the aged MPs produced by the mixer-milling process had a statistically higher density than those produced by ultracentrifugal milling. This increase is also likely due to the more fragmented and irregular surfaces of the MPs produced by mixer milling, which may have facilitated the incorporation of biofilm and microbial exudates, thereby increasing the overall density of the particles, which is consistent with our previous results.^31^

Crystallinity analysis was performed only for PP, as PS was amorphous. For PP, the changes in crystallinity were consistent with the extent of biofilm accumulation. Mixer-milled PP MPs, which supported more extensive biofilm growth, exhibited a lower degree of crystallinity than those produced by an ultracentrifugal mill. In contrast, the crystallinity of ultracentrifugally milled PP remained close to that of pristine PP, with only a slight reduction due to minimal biofilm coverage. Since biofilms are inherently amorphous,^50,51^ their presence contributes to an overall decrease in the crystallinity of MPs.

The results of this study have important consequences for MP environmental research. The observed differences in biofilm formation and microbial colonization highlight the importance of selecting fragmentation methods for the preparation of MPs for environmental research. Therefore, fragmentation techniques may influence not only the morphology and physicochemical properties of MPs but also their interactions with biotic components in aquatic ecosystems. These fragmentation-induced differences arise before environmental exposure and define the initial physicochemical conditions under which microbial colonization and biotic aging take place. Consequently, the fragmentation method should be regarded as an integral component of the MP experimental design rather than merely a procedure for producing particles within a desired size range. MPs with higher irregularity, such as those produced by mixer milling, may experience a greater degree of biotic aging. However, this effect is not only due to surface morphology and texture; surface chemistry, polymer type, and the type of biofilm formation also play a crucial role. Biofilm accumulation and selectivity could be influenced by changes in functional groups and microbial interactions, which depend on both the physical properties and the chemical reactivity of the particles. The surface changes induced by milling methods, combined with polymer-specific behaviors, contribute to variations in the long-term fate and behavior of MPs in the natural environment, affecting their persistence, bioavailability, and ecological risks.

At the same time, it is important to emphasize that these findings apply to the specific polymers tested (PS and PP), the fragmentation techniques employed, and the freshwater aging conditions used in this study. The conclusions of the present study are based on multiple measurements performed on large ensembles of particles produced by using each fragmentation technique, providing a robust statistical basis for the observed trends. However, batch-to-batch variability between independently produced MP preparations was not explicitly reassessed in the present study and should, therefore, be considered a potential limitation. Extrapolation to other polymer types, environmental compartments, or degradation pathways should therefore be made with caution.

Furthermore, the study underlines the critical need to select suitable test materials for the laboratory activities. The comparability of results can be significantly influenced by the physicochemical properties of the test materials themselves, and overlooking this factor could result in incorrect interpretations of the environmental behavior of MPs, as variations in surface chemistry and morphology can affect microbial interactions and subsequent aging processes.

## Supplementary Material



## Data Availability

Experimental data and measurements regarding biofilm formation and changes in crystallinity and density are publicly available in the Repository of the University of Ljubljana under the persistent identifier: 20.500.12556/RUL-181469. Other data will be made available on request.

## Notes

Methodological details on the microplastics investigated in this study; microplastic size distribution data; a description of the procedure used to determine morphological descriptors of microplastics, together with the resulting data, and a description of the wettability measurements and their results (PDF)
