# Supplementary material for: Influence of Fragmentation Techniques on the Biotic Aging of Test Microplastics
Source: Environ Sci Technol. 2026 Aug 28;60(36):25701–15. doi: 10.1021/acs.est.6c03742 (PMC13585064; doi:10.1021/acs.est.6c03742)
Supplement: Supplementary file 1 [file es6c03742_si_001.pdf]

Supporting Information

Influence of fragmentation techniques on the biotic aging of test microplastics

Barbara Klun[1]#, Serena Ducoli[2]#, Mark Starin[1], Janja Novak[1], Filippo Vaccari[3], Edoardo Puglisi[3], Ula Rozman[1], Nataša Čelan Korošin[1], Gabriela Kalčíková[1]*, Stefania Federici[2]*

[1]Faculty of Chemistry and Chemical Technology, University of Ljubljana, Večna pot 113, 1000 Ljubljana, Slovenia

[2]Department of Mechanical and Industrial Engineering, University of Brescia & INSTM RU of Brescia, Via Branze 38, 25123 Brescia, Italy

[3] Università Cattolica del Sacro Cuore, Department for Sustainable Process, Faculty of Agriculture, Food and Environmental Science (DiSTAS), Via Emilia Parmense 84, 29122 Piacenza, Italy

#Authors contributed equally to the work and are both co-first authors.

S1.1 Particle size distribution

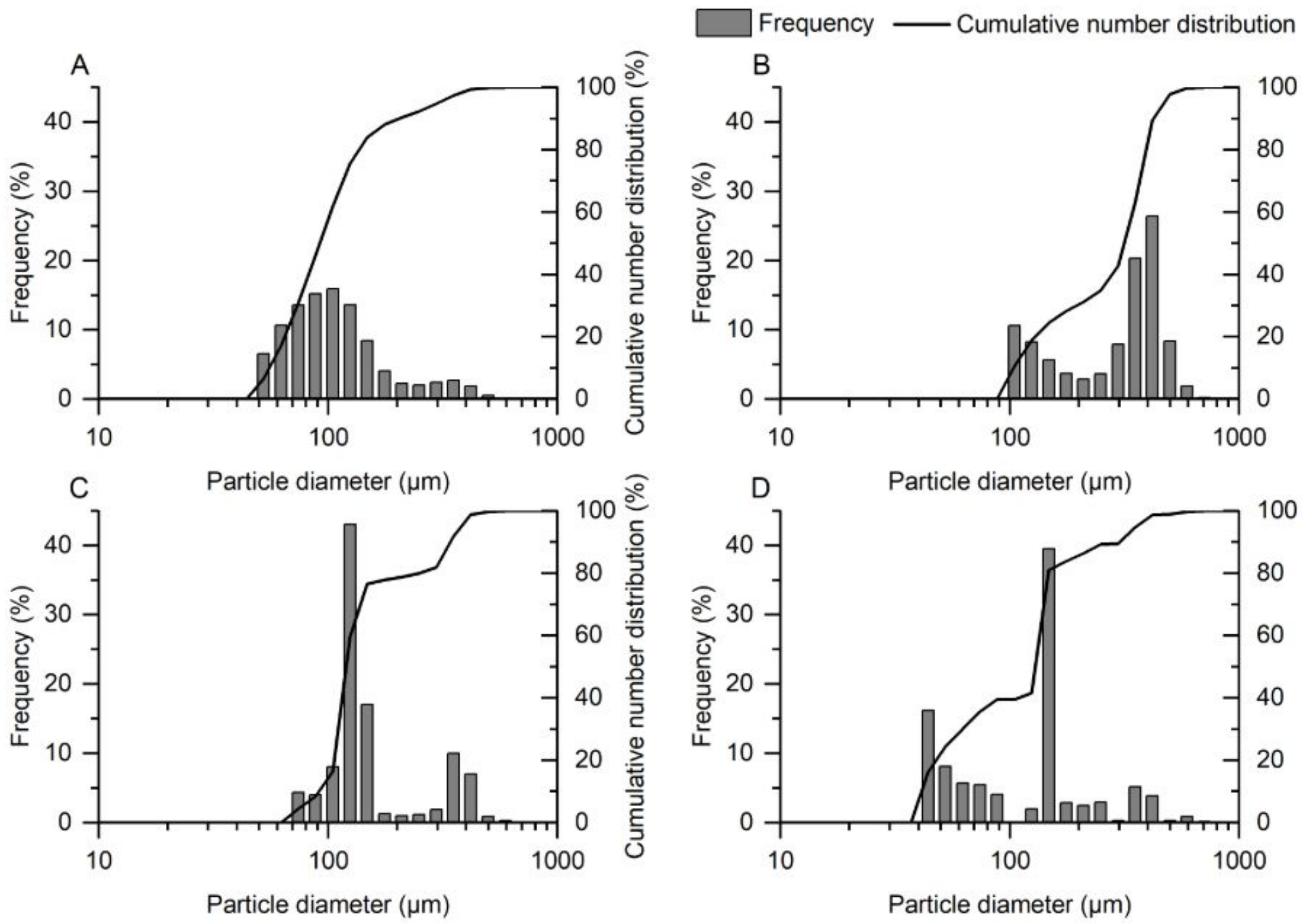

Figure S1: Particle size distribution of pristine polystyrene (PS) and polypropylene (PP) microplastics prepared with different fragmentation techniques: A) mixer-milled PP, B) ultracentrifugally milled PP, C) mixer-milled PS and D) ultracentrifugally milled PS.

S1.2 Morphological descriptors of pristine microplastics

MP morphological descriptors were calculated based on optical microscopy and image analysis. For each MP class (mixer-milled PP, ultracentrifugally milled PP, mixer-milled PS, and ultracentrifugally milled PS), high-magnification images of approximately 50 particles were acquired with a stereo microscope equipped with an HD Digital Camera (MZ16-A and MC190, Leica Microsystems, Germany). Leica Image analysis software (Leica Application Suite (LAS) 4 software enhanced with Interactive Measurements and Image Analysis modules) was employed to get shape descriptors from each particle, particularly perimeter (mm), convex perimeter (mm), area (mm^2), and convex

area (mm²) (Figure S2). Particle edge irregularity was quantified through the convexity of the particle, defined as the ratio between the perimeter of the particle's convex hull and the actual perimeter of the particle itself (convex perimeter · perimeter^{-1}). Solidity was also calculated as the ratio between the area of the particle and the area of the particle's convex hull (area · convex area^{-1}).

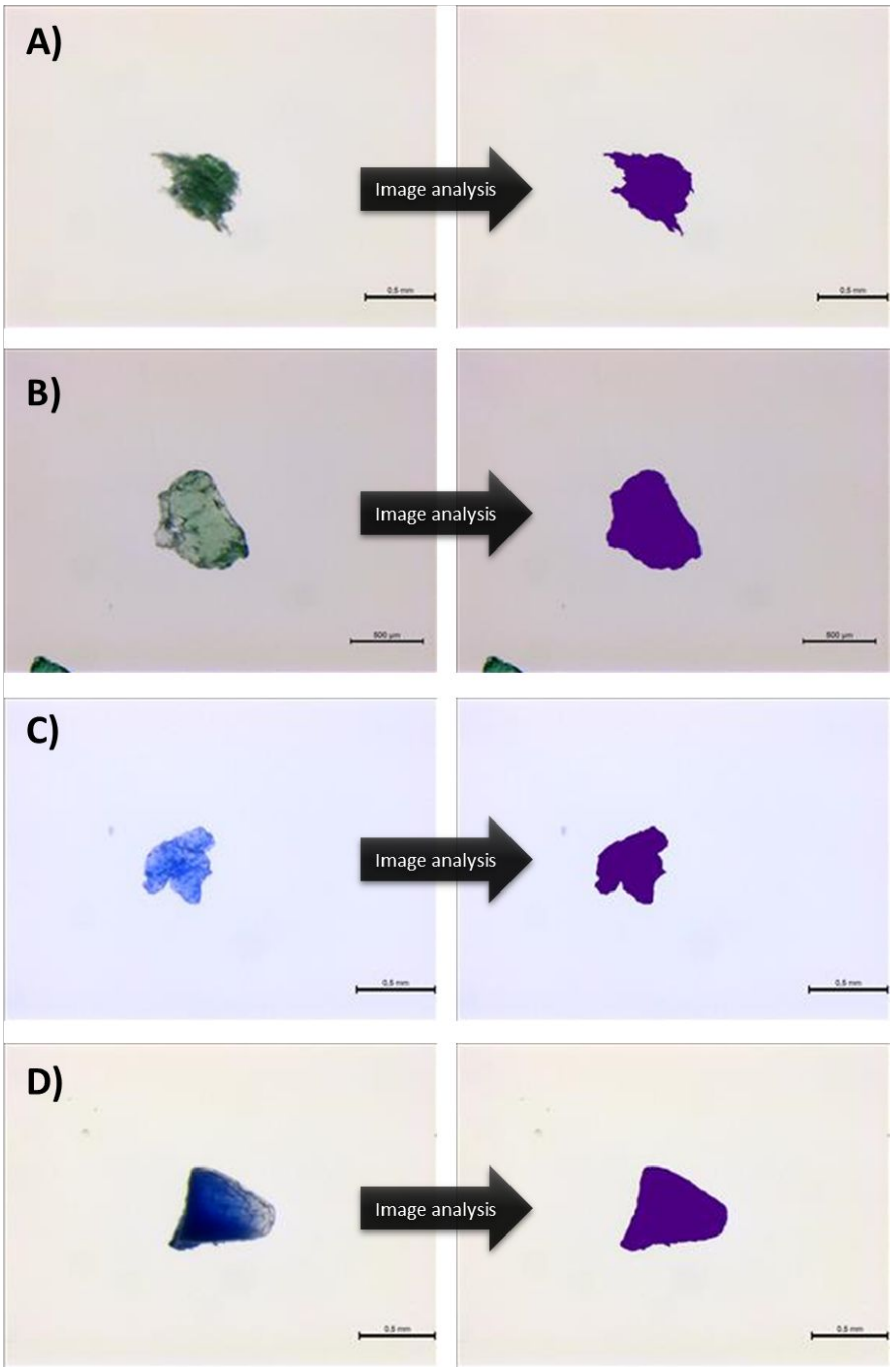

Figure S2: Examples of optical microscopy images of pristine polystyrene (PS) and polypropylene (PP) microplastics prepared with different fragmentation techniques: A) mixer-milled PP, B) ultracentrifugally milled PP, C) mixer-milled PS, and D) ultracentrifugally milled PS; before (left) and after (right) image analysis.

The convexity of ultracentrifugal milled MPs was statistically higher compared to the convexity of mixer-milled MPs (Figure S3), for both PP MPs (p value = $1.9 \cdot 10^{-22}$) and PS MPs (p value = $4.9 \cdot 10^{-7}$). These differences indicate that MPs produced by mixer milling exhibit a higher degree of particle edge irregularity than MPs fragmented by ultracentrifugal milling.

Interestingly, the solidity of the same particles resulted in no statistically significant difference between the varying fragmentation classes (Figure S4). The convexity measures how irregular the edge of the particle is, while the solidity measures how well the particle fills its convex envelope, being therefore especially sensitive to the presence of large concavities that reduce the occupied area. These results suggest that the fragmentation methods primarily affect the complexity of the particle outline, without inducing substantial changes in the overall particle shape or the presence of large concavities.

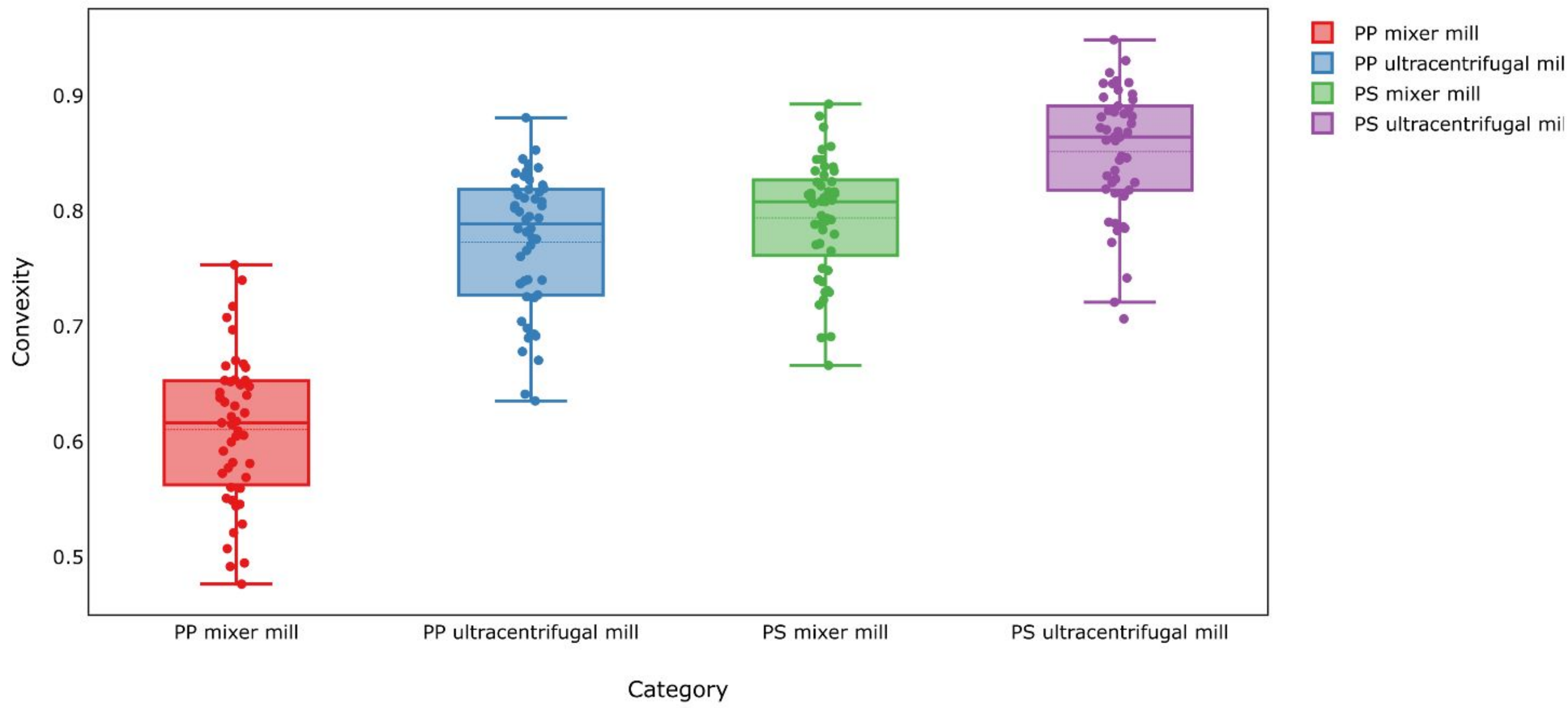

Figure S3: Convexity of pristine polystyrene (PS) and polypropylene (PP) microplastics fragmented using either ultracentrifugal mill or mixer mill.

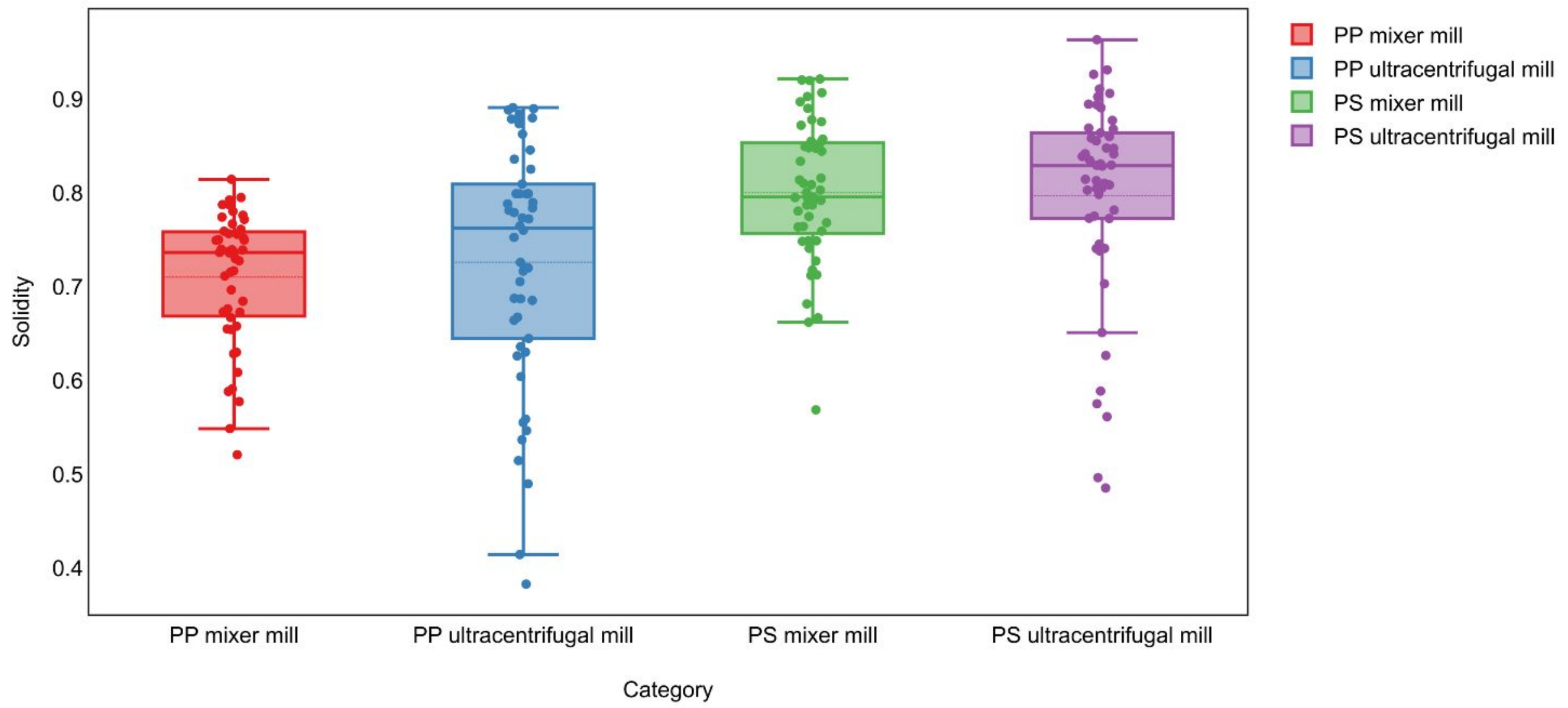

Figure S4: Solidity of pristine polystyrene (PS) and polypropylene (PP) microplastics fragmented using either ultracentrifugal mill or mixer mill.

S1.3 Wettability of pristine microplastics

Contact angle measurements were performed on compressed MP pellets to evaluate MP wettability. Approximately 150 mg of MPs were compressed using a hydraulic press under 200 bar of pressure for a few minutes to create compact, flat MP pellets. Pellets of MPs were let equilibrate in air for 24 hours before the analysis. Five drops of milliQ water were deposited on the pellet surface, and the time evolution of the droplet profile was acquired using a high-resolution digital camera (CAM 200 tensiometer (KSV Instruments, Finland). The software calculates the angle formed between the solid surface and the liquid tangent line. For each MP class, three pellets were prepared.

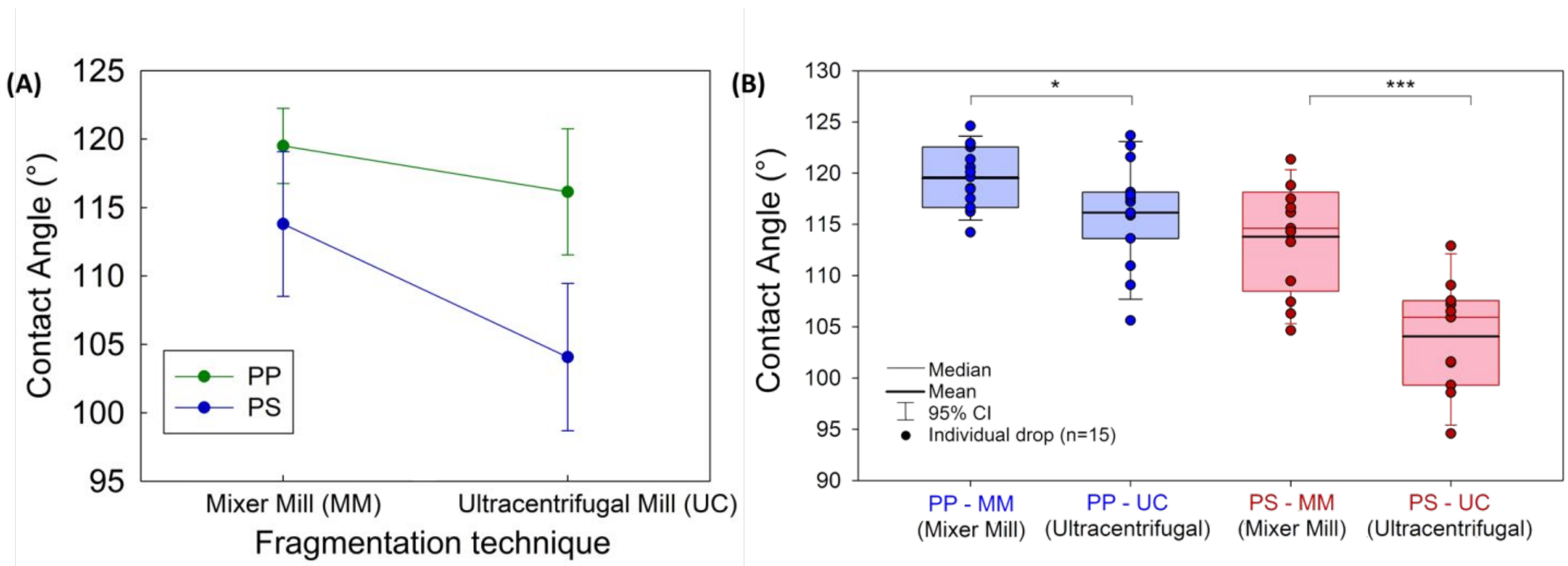

Figure S5: Apparent contact angle of microplastic pellets. A) Interaction plot showing the effect of fragmentation technique (ultracentrifugal mill (UC) vs. mixer mill (MM)) on the contact angle for polypropylene (PP, green) and polystyrene (PS, blue) microplastics. Points represent mean values, and error bars indicate the standard deviation over replicates. B) Boxplot with individual measurements (15 drops per MP class: 3 pellets x 5 drops). Boxes show the interquartile range with median (line), means (thick line), and 95% confidence interval (CI) of the mean. Asterisks indicate statistically significant differences between fragmentation methods for the same polymer (*$p < 0.05$; ***$p < 0.001$).

Because of these different fragmentation mechanisms, the resulting MPs exhibited distinct physicochemical characteristics already before biotic aging, including particle size distribution, particle morphology, and apparent wettability. Contact angle measurements performed on compacted MP pellets showed that mixer-milled MPs exhibited significantly higher apparent contact angles than ultracentrifugally milled MPs for both polymers (Figure S5A), indicating that the fragmentation technique also modifies the interfacial properties of the resulting particles. The effect was particularly pronounced for PS (contact angle values of 114 ± 5 for mixer mill and 104 ± 5 for ultracentrifugal mill), whereas a smaller but still significant increase was observed for PP (contact angle values of 120 ± 3 for mixer mill and 116 ± 4 for ultracentrifugal mill). Although the measured values represent the apparent wettability of compacted particle beds rather than the intrinsic contact angle of the polymer surface, they consistently demonstrate that the fragmentation process generates MPs with distinct surface/interfacial characteristics prior to environmental exposure.
